# Exposure to toxic metals triggers unique responses from the rat gut microbiota

**DOI:** 10.1038/s41598-018-24931-w

**Published:** 2018-04-26

**Authors:** Joshua B. Richardson, Blair C. R. Dancy, Cassandra L. Horton, Young S. Lee, Michael S. Madejczyk, Zhenjiang Zech Xu, Gail Ackermann, Gregory Humphrey, Gustavo Palacios, Rob Knight, John A. Lewis

**Affiliations:** 1 Oak Ridge Institute for Science and Education, Fort Detrick, MD 21702 USA; 2 United States Army Center for Environmental Health Research, Fort Detrick, MD 21702 USA; 3 Center for Genome Sciences, United States Army Medical Research Institute of Infectious Diseases, Fort Detrick, MD 21702 USA; 4 Department of Pediatrics, University of California, San Diego, La Jolla, CA 92093 USA; 5 Department of Computer Science and Engineering, University of California, San Diego, La Jolla, CA 92093 USA; 6 Center for Microbiome Innovation, University of California, San Diego, La Jolla, CA 92093 USA

## Abstract

Our understanding of the interaction between the gut microbiota and host health has recently improved dramatically. However, the effects of toxic metal exposure on the gut microbiota remain poorly characterized. As this microbiota creates a critical interface between the external environment and the host’s cells, it may play an important role in host outcomes during exposure. We therefore used 16S ribosomal RNA (rRNA) gene sequencing to track changes in the gut microbiota composition of rats exposed to heavy metals. Rats were exposed daily for five days to arsenic, cadmium, cobalt, chromium, nickel, or a vehicle control. Significant changes to microbiota composition were observed in response to high doses of chromium and cobalt, and significant dose-dependent changes were observed in response to arsenic, cadmium and nickel. Many of these perturbations were not uniform across metals. However, bacteria with higher numbers of iron-importing gene orthologs were overly represented after exposure to arsenic and nickel, suggesting some possibility of a shared response. These findings support the utility of the microbiota as a pre-clinical tool for identifying exposures to specific heavy metals. It is also clear that characterizing changes to the functional capabilities of microbiota is critical to understanding responses to metal exposure.

## Introduction

The microbiota of the mammalian gut is recognized as an important factor in maintaining host health^1^. For example, the gut microbiota is crucial for the development of the immune system and a healthy gastrointestinal tract^2,3^. Disruption of an established gut microbiome is associated with diseases ranging from obesity^4^, diabetes^5^, and allergies^6^ to intestinal bowel disease^7^. It is therefore critical to understand the factors that can disrupt or alter the microbiota. Factors such as stress, diet and genetics are known to affect the microbiota, and, increasingly, studies indicate a role for environmental toxicants as well^8^. Toxicants in the environment can directly harm the components of the microbiota, but can also be modified by the microbiota to be more or less toxic to the host and/or the microbiota itself^9^. The microbiota of the gut occupies a niche at the interface of the external environment and host epithelium which makes it a prime target for monitoring environmental exposures. If there are taxa especially sensitive to environmental pollutants, monitoring changes to the microbiota could yield biomarkers of exposure.

Several studies have begun to explore how different environmental toxicants, such as metals, interact with the microbiota specifically^8–11^. Metals can be highly reactive, and both prokaryotes and eukaryotes have evolved mechanisms to take advantage of and to protect themselves from exposure. For example, the reactivity of some metals, like iron, manganese, and cobalt, make them useful as cofactors in enzymes to help catalyze reactions. In addition, there are a number of cellular transport systems, binding proteins, and conjugation pathways to limit exposures and unwanted effects from both essential and non-essential metals. The doses necessary to overwhelm these homeostatic mechanisms vary by organism and environment, and levels that may be toxic to some may have no, or even a beneficial effect, on others. As the microbiota experience the exposure prior to the host, how those microbes react to or affect the exposure has the potential to influence the host response.

Metal exposures are an environmental and occupational hazard. The metals selected for this work, chromium (Cr), cadmium (Cd), cobalt (Co) and nickel (Ni), are widely distributed and some of the most utilized metals in industry, while arsenic (As) is a persistent public health threat found at high concentrations in drinking water in many areas^12^. All five of these metals have been listed by IARC as Group I or II carcinogens^13,14^. In addition, exposure can lead to adverse health effects in target organs such as the liver, kidney, and lungs, although they are thought to act via different mechanisms and biochemical pathways^14–16^.

Exposures to metal compounds have been shown to alter the diversity and composition of the gut microbiota. For example, in one study, either cadmium or lead in drinking water alter mouse gut microbiota by increasing Lachnospiraceae abundance^11^, while a different study found that cadmium in water increased growth of Bacteroidetes relative to Firmicutes in mice^17^. Another mouse study found that cadmium exposure led to a relative increase in Verrucomicrobia^18^. Chickens fed nickel-supplemented feed had increased prevalence of *E. coli* and *Enterococcuss* species^19^. Studies examining alterations in the microbial composition after arsenic exposure have shown results that appear to conflict. Guo *et al*. found that providing mice water containing arsenic increased the abundance of Firmicutes and decreased the abundance of Bacteroidetes^20^. However, Dheer *et al*. found that arsenic exposure caused an increase in Bacteroidetes and a decrease in Firmicutes^21^. In another mouse study, drinking arsenic-containing water caused a decrease in the prevalence of the class Clostridia (in the phylum Firmicutes)^22^. Most studies use the animal’s food or water as the source of exposure, which can lead to variable dosing of individuals. In addition, these studies tend to look at changes after long periods of exposure, potentially missing important early changes to the microbiota.

**Figure 1:**
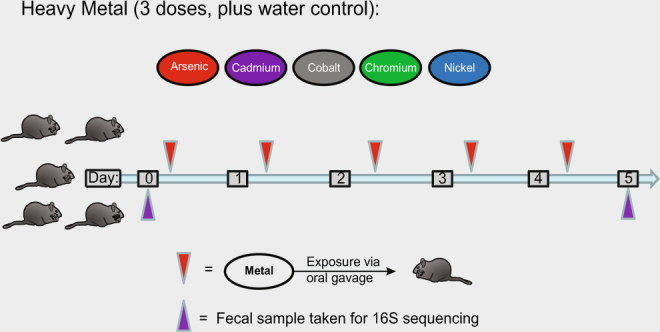
Experimental summary. Diagram of dosing and sample schedule. A cohort of 5 rats were exposed to one of the indicated metals at a particular dose for five days, with samples taken before and after the exposures, as shown.

To gain a more comprehensive understanding of the effects of metal exposure on the gut microbiota, we exposed rats to three different oral doses of sodium arsenite, cadmium chloride, cobalt chloride, sodium dichromate, or nickel chloride for five days (Fig. 1). We profiled the gut microbiota composition using 16S rRNA gene sequencing before and after exposure to identify the taxa most sensitive or resistant to the metal. We identified taxa and inferred gene inventories that are enriched or depleted after metal exposure, detailing how each metal altered the microbiota. This information is likely to prove useful from a health monitoring standpoint if some taxa can serve as indicators of low/sub-clinical exposures that are not immediately harmful but could become so over time. Further, this design allowed us to identify early responses to metal exposures, such as enrichment of certain taxa or genes, which could be beneficial to the host and/or the microbiota in adapting to the higher levels of metal and that could be exploited to craft a therapeutic strategy.

## Results

Cohorts of five rats were exposed to three different doses of sodium arsenite, cadmium chloride, sodium dichromate, cobalt chloride, or nickel chloride by oral gavage for five consecutive days. Fecal pellets were collected before the initial exposure and 24 hours after the final exposure. DNA from the fecal pellets was isolated, and the V4 region of the 16S rRNA gene was amplified using degenerate primers to the flanking conserved regions and sequenced on an Illumina MiSeq. We recovered 14,703,356 reads from one multiplexed run containing the sequencing libraries for all of the samples. After joining paired-end reads, 11,127,881 sequences remained. Demultiplexing, quality filtering, and removal of chimeric sequences left 8,357,686 sequences. Samples with less than the minimum reads for inclusion (3,544, see methods) were excluded from the analysis. This left 279 samples, with an average of 15,317 sequences/sample.

### Overview of Changes in Microbiome Composition

Reads were clustered into operational taxonomic units (OTUs) based on 97% similarity using the QIIME pipeline and assigned a taxonomic designation using the Greengenes v13.8 database. Across the entire data set, 99% of the OTUs could be assigned to a phylum, 86% could be assigned to a family, and 39% could be assigned to a named genus. The drop-off in assignments at the genus level is in part due to the large percentage of OTUs belonging to the S24-7 family, which does not have a clearly resolved phylogeny below the family level and consequently does not have associated genera in the Greengenes v13.8 database^23^.

**Figure 2:**
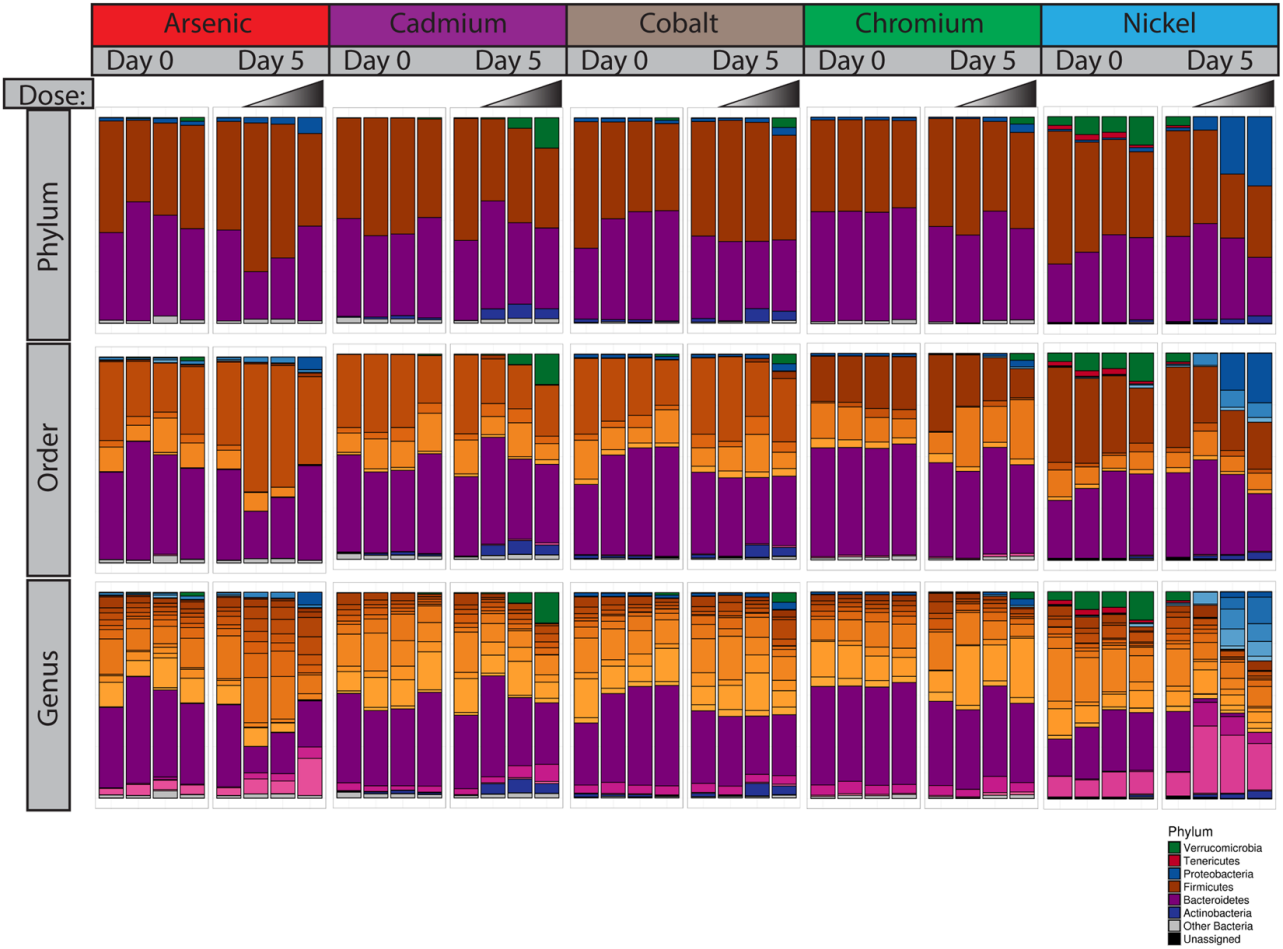
Taxonomic summaries. Stacked bar plots showing the average relative abundance of each taxa at various taxonomic levels. Different colored bars represent different phyla (indicated by the key), and, for the Order and Genus level plots, different tones represent different order and genera within the specified phylum. Only phyla with an abundance of >5% in at least one sample of the exposure are shown. Any other phyla are classified as “Other Bacteria.”

Figure 2 summarizes the abundances of taxa at the phylum, order and genus levels. The pre-exposure samples (Day 0) and post-exposure control samples (water control) have generally similar proportions of the major phyla. The cohorts all have roughly equal amounts of Bacteroidetes and Firmicutes, which together comprise on average 92% of the total bacteria across all samples and are known common components of the mammalian gut microbiota^24^. A small amount of Proteobacteria and Actinobacteria is also present in most controls. Notably, the samples from the nickel cohort (including pre-exposure and control samples) are well distinguished from the other control samples by the presence of Verrucomicrobia and Tenericutes, which collectively make up around 10% of the bacteria in the nickel control samples (see discussion for further details).

**Figure 3:**
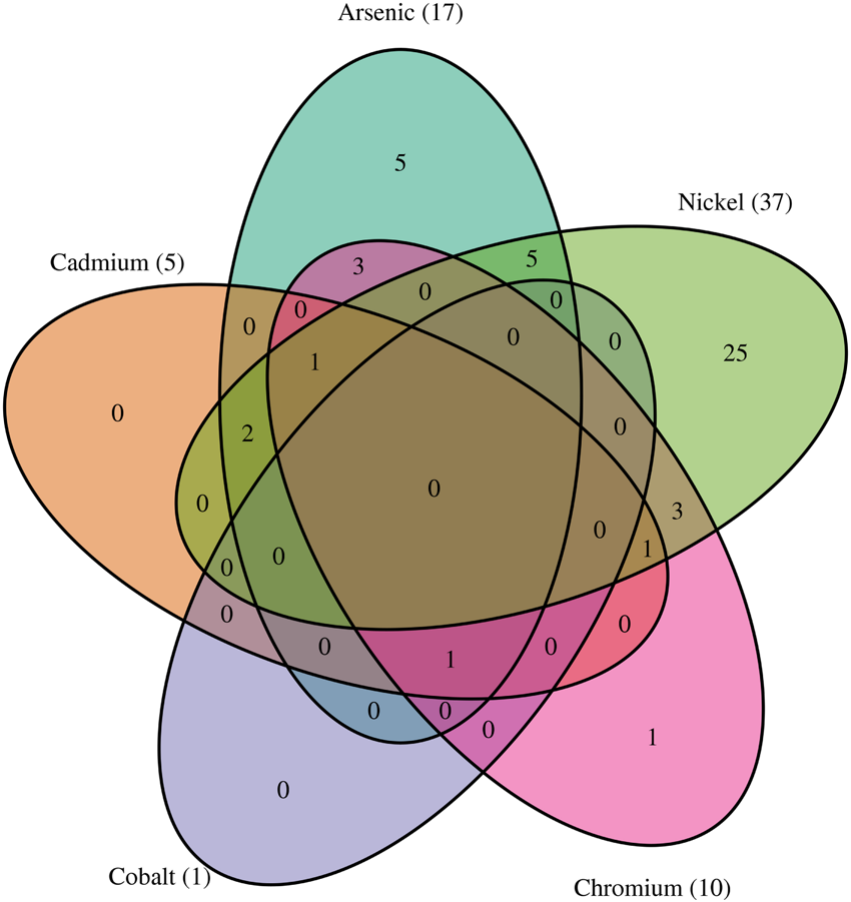
Venn diagram showing genera that are significantly different in abundance before and after metal exposure. Significance tested by the Wald test (p ≤ 0.05). See Fig. 6 and Supplementary Table S1 for taxa names.

Phylum level changes in the gut microbiota were induced to varying degrees by each of the metals tested, particularly at the highest dose level. Proteobacteria increased in prevalence in response to each metal except for cadmium. Verrucomicrobia increased in cadmium, cobalt and chromium exposed samples despite not being a major presence in the control samples for these metals. In contrast, the Verrucomicrobia in the nickel control samples appear to have been reduced after exposure. The nickel exposure also seems to have nearly eliminated the Bacteroidetes family S24-7, which is the most prevalent Bacteroidetes family in the other samples and is prevalent in rat gut microbiota generally^23^. Interestingly, the decline of family S24-7 in nickel-exposed samples was accompanied by an increase in the presence of other non-S24-7 Bacteroidetes. Proteobacteria, specifically bacteria belonging to the Enterobacteriaceae family, also greatly increased after nickel exposure. Overall, the pattern of changes in microbiome composition after metal exposure tended to be specific to a particular metal, though the majority of the genera affected (with the exception of those identified in the nickel exposures) had altered abundances in multiple metals (Fig. 3). While there were some qualitatively similar changes, such as an increase in Proteobacteria, the scale of the change varied between metals, and no consistent patterns at any taxonomic level were observed across all metals.

### Alpha Diversity

**Figure 4:**
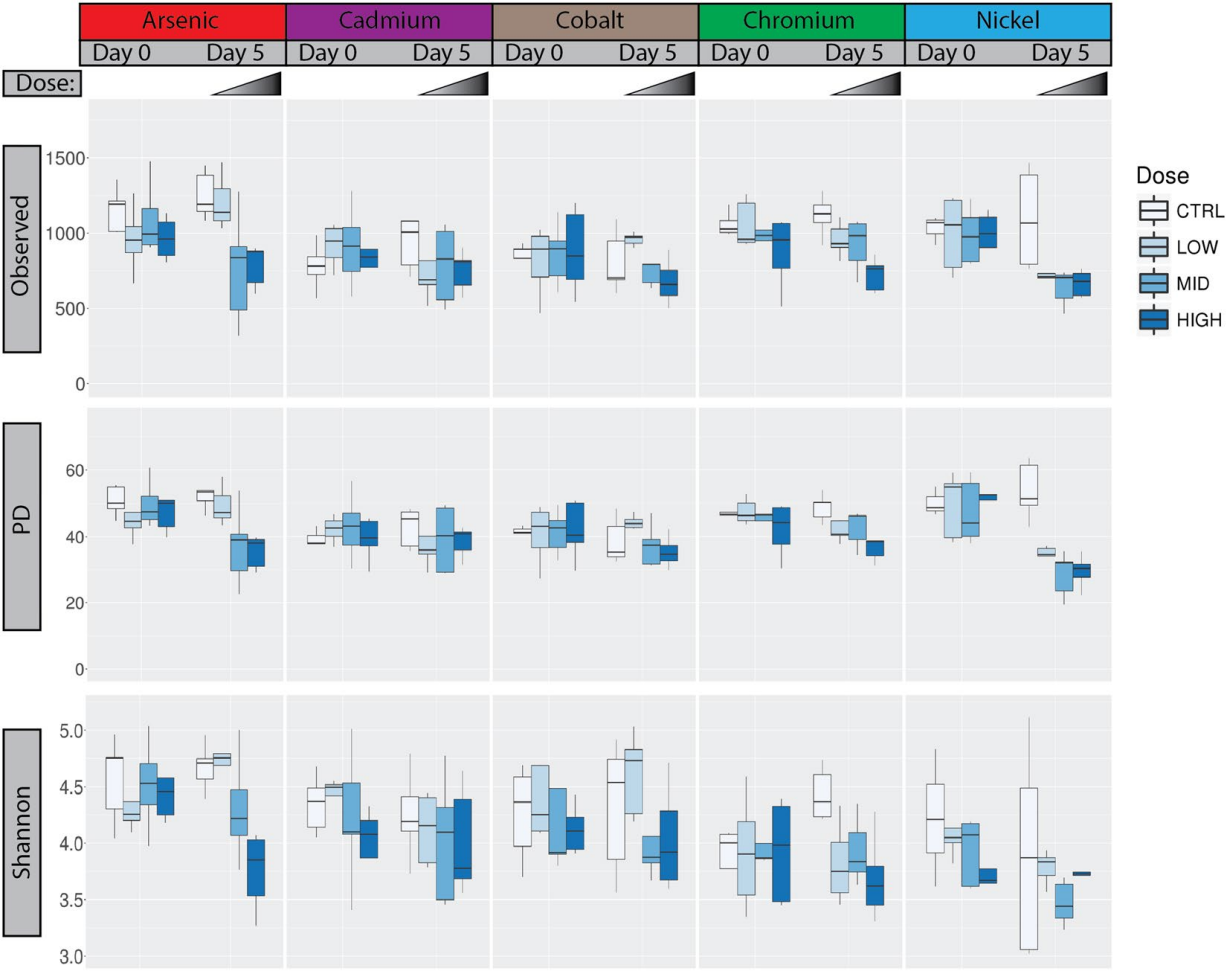
Alpha Diversity. Top row: Number of OTUs observed in each cohort pre- (D0) and post-exposure (D5) for the indicated metal. Middle row: Faith’s Phylogenetic Diversity (PD) pre (D0) and post-exposure (D5). Bottom row: Shannon’s diversity index for each cohort pre- (D0) and post-exposure (D5) for the indicated metal. In each plot, color indicates dose level.

Plots of the within sample (or “alpha”) diversity across cohorts (Fig. 4, top row) show the average number of OTUs observed in samples pre- and post-exposure. Samples taken from animals after exposure to arsenic, chromium, and nickel trended towards lower numbers of observed OTUs relative to their respective post-exposure control. Similar results were observed using Faith’s phylogenetic distance (PD) and Shannon’s diversity, an alpha diversity metric that incorporates the evenness and abundance of different OTUs (Fig. 4). One-way analysis of variance (ANOVA) indicates dose is a significant factor determining observed OTUs and PD among day 5 arsenic, chromium and nickel samples *(p* < 0.05). Dose was also a significant contributor to Shannon’s diversity in arsenic and chromium samples (*p* < 0.05). Despite reduced alpha diversity metrics in the arsenic, chromium and nickel treatments, the large within treatment variability makes firm conclusions difficult.

### Beta Diversity

**Figure 5:**
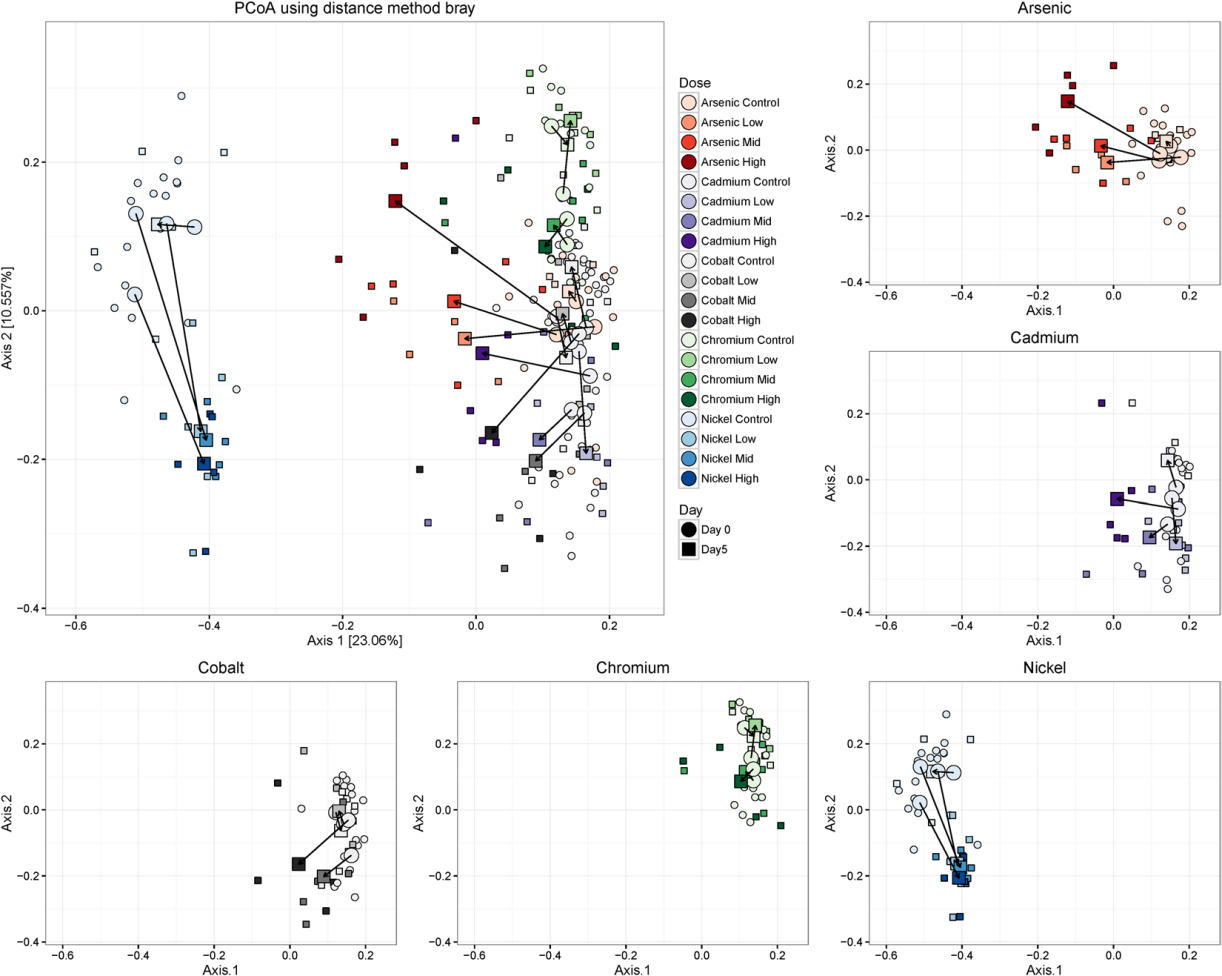
Principal Coordinate Analysis using the Bray-Curtis distance metric. The main figure shows first two coordinate axes from a Principal Coordinate Analysis based on the bray distance. Shape indicates day of sampling. Color indicates type of metal exposure, and shading indicates dose level. The small shapes indicate individual samples, and large shapes indicate centroids. Arrows connect the pre- and post-exposure centroids for each cohort.

As another means of identifying changes in the microbiota induced by metal exposure, we performed a principal coordinate analysis (PCoA) of all samples in our study using the Bray-Curtis distance metric on OTUs identified by QIIME (Fig. 5). Axis 1 captures 23% of the total variance, separating the entire nickel cohort from the remaining samples, and separating the post-exposure samples from pre-exposure samples for the other metals. Axis 2 separates the pre-exposure samples from each other (apart from nickel) and separates the post- from pre-exposure nickel samples. The nickel cohorts, including the pre-exposure samples are well separated from the rest of the samples along axis 1. Pre- and post-exposure samples from different metals tended to cluster apart, emphasizing the uniqueness of responses to different metal exposures. However, exposure caused a shift along axis 1 in the same direction for each metal except for nickel, suggesting some aspects of the response is shared. One potential candidate is the phylum Verrucomicrobia, which increases in abundance after exposure to cadmium, cobalt and chromium. A qualitatively similar result was obtained using the UniFrac distance method, which incorporates phylogenetic information when determining distance between OTUs (see Supplementary Fig. S1)^25^.

We tested for significant differences in beta diversity between pre- and post-exposure samples within a cohort, and between post-exposure controls and treated samples using the PERMANOVA test on OTU tables. The PERMANOVA results (R^2^ (effect size) and *p* values) are shown in Table 1. Because the PERMANOVA test is sensitive to differences in variance, we performed a multivariate test for equality of variances using the “betadisper” function from the vegan R package, in addition to the PERMANOVA. Only three comparisons (out of 35) showed a significant result (p < 0.05, Table 1, “Betadisper P”), indicating most significant PERMANOVA results are due to a change in mean and not variance. For each of the metals, the differences in pre- and post-exposure water controls were not significant (Table 1, column 3), suggesting no effect due to experimental manipulation (gavage). Each post-exposure treatment group from the arsenic, cadmium and nickel experiments were significantly different from the post-exposure control group (Table 1, column 1). Only the chromium high dose group was significantly different from the chromium control, and no significant differences were observed due to cobalt exposure. The size of exposure effects (R^2^) varied between treatments, with the largest effect seen in the nickel high dose treatment group (R^2^ = 0.55), and the smallest significant effect seen in the chromium high dose treatment group (R^2^ = 0.22). In addition, we tested if metal treatments showed a significant, dose-dependent effect on microbiota composition, where each dose must induce a significant change from lower doses, and not just a change from the control (see methods for details). Arsenic, cadmium, and nickel all show dose dependent effects at each dose (Table 1, column 2). In summary, cobalt and chromium treatments showed little effect on microbiota composition, while arsenic, cadmium and nickel significantly altered microbiota composition in a dose-dependent manner.

**Table 1:** Treatment effects on beta diversity.

Metal	Dose	Exposed vs Sham Control			Dose Effect		Pre- vs Post-Exposure		
		Equal Variance^a^	Effect Size^b^	*P* ^c^	Effect Size^b^	*P* ^c^	Equal Variance^a^	Effect Size^b^	*P* ^c^
Arsenic	Control	NA			NA		0.414	0.087	0.750
	Low	0.957	0.376	**0.008**	0.133	**0.002**	0.295	0.458	**0.008**
	Mid	**0.001**	0.268	**0.007**	0.066	**0.045**	0.236	0.257	**0.009**
	High	0.187	0.443	**0.006**	0.192	**0.001**	0.262	0.454	**0.006**
Cadmium	Control	NA			NA		0.258	0.115	0.417
	Low	0.602	0.245	**0.016**	0.111	**0.004**	0.989	0.193	0.061
	Mid	0.237	0.352	**0.007**	0.117	**0.003**	0.478	0.264	**0.014**
	High	0.684	0.373	**0.009**	0.143	**0.003**	0.559	0.393	**0.007**
Cobalt	Control	NA			NA		0.798	0.083	0.736
	Low	0.947	0.070	0.905	0.029	0.957	0.668	0.082	0.796
	Mid	0.709	0.157	0.128	0.088	0.062	0.576	0.146	0.166
	High	0.281	0.172	0.093	0.084	0.103	0.098	0.212	**0.015**
Chromium	Control	NA			NA		0.417	0.182	0.135
	Low	0.393	0.155	0.147	0.064	0.188	0.9	0.203	**0.050**
	Mid	0.853	0.158	0.137	0.077	0.068	0.261	0.151	0.145
	High	0.937	0.215	**0.007**	0.100	**0.023**	0.423	0.204	**0.008**
Nickel	Control	NA			NA		0.213	0.146	0.390
	Low	0.110	0.533	**0.007**	0.244	**0.001**	**0.023**	0.594	**0.007**
	Mid	0.456	0.457	**0.008**	0.139	**0.004**	0.001	0.539	**0.009**
	High	0.446	0.553	**0.008**	0.138	**0.007**	0.602	0.629	**0.007**

Results from the PERMANOVA test on each cohort, comparing post-exposure control samples to post-exposure treated samples (“Exposed vs Sham Control”). Dose-dependent effects are shown under “Dose Effects.” PERMANOVA tests comparing pre and post-exposure samples are shown under “Pre- vs Post-Exposure.” ^a^Results of a multivariate test of equality of variances using the “Betadisper” function from the vegan R package. Statistical significance indicates that the variances are not equal. ^b^The proportion of variance explained by exposure status. ^c^The probability that both sample groups occupy the same location (have similar microbiome compositions) based on the Bray-Curtis distance metric using the PERMANOVA test. P values less than 0.05 are in bold.

### Differential Abundance of Taxa

**Figure 6:**
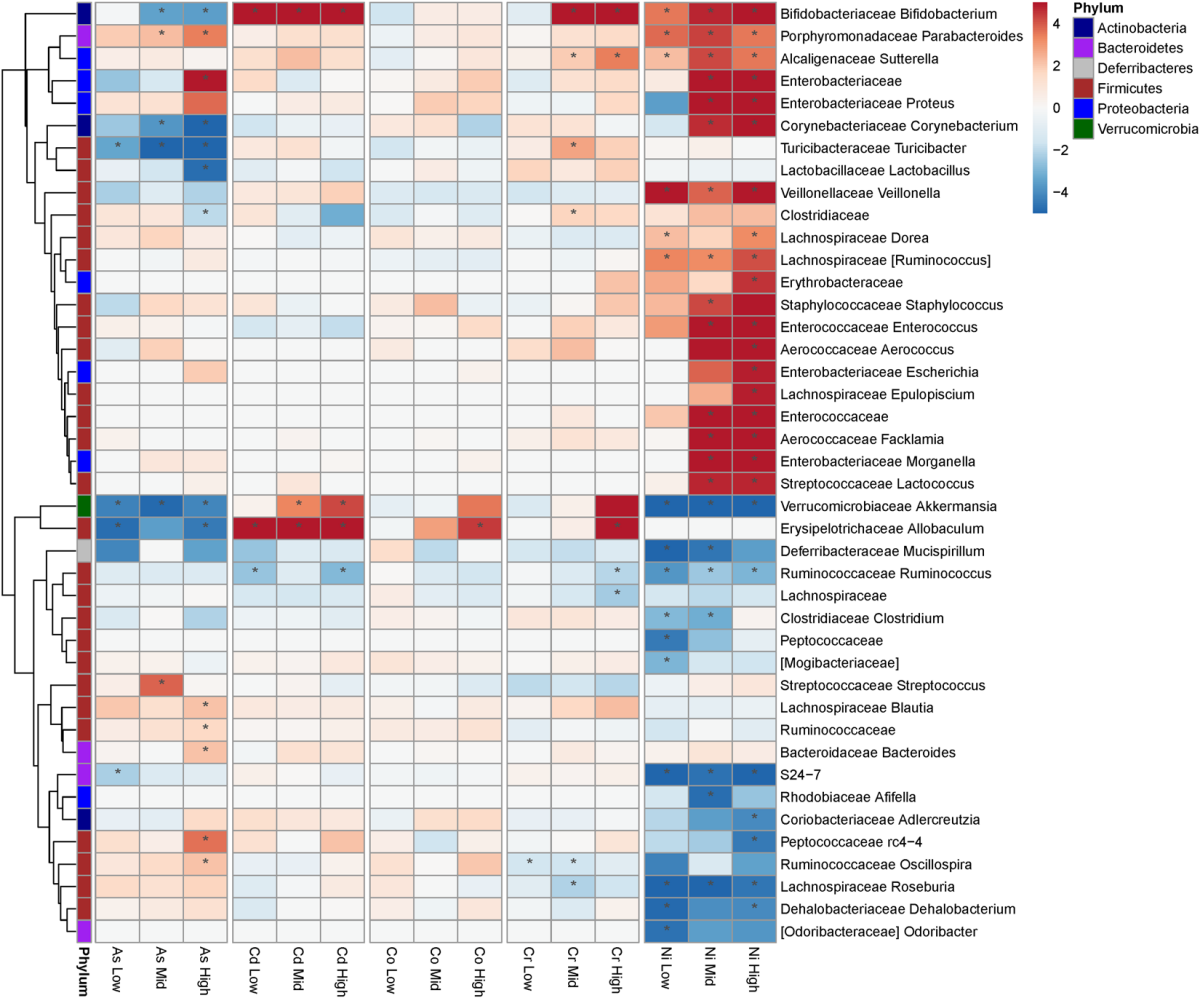
Heat map showing the average log-fold change relative to sham controls. Red cells indicate increased abundance due to treatment. The phylum is indicated by bars to the left. Only taxa that were significantly changed in at least one test are shown. Only the family and genus are shown, see Supplementary Table S1 for complete taxonomy. *Indicates significant association of exposure with change in abundance (Wald test) at p ≤ 0.05.

We identified taxa with different abundances in post-exposure treated samples relative to vehicle controls. Figure 6 shows a heat map of all genera that had significantly different abundances (Wald test, p < 0.05) in at least one comparison. In total, the abundances of 47 genera were affected by at least one metal exposure. Exposure to nickel showed the most pronounced effect with changes in abundance to 37 genera, of which 25 were uniquely affected by this metal. In contrast, only one genus was identified as changing in the cobalt cohort. Figure 3 shows the overlap of significantly different genera among the metals.

### Differential Abundance of Genes and Gene Pathways

We estimated the relative abundance of genes and biological pathways using the PICRUSt tool (phylogenetic investigation of communities by reconstruction of unobserved states^26^). This tool estimates the gene content of a bacterial assemblage based on the bacteria identified by 16S rDNA sequencing. Pathways, based on KEGG annotations, are then identified using this inferred gene inventory. The nearest sequenced taxon index (NSTI), a measure of PICRUSt accuracy, was 0.168+/− 0.059 (mean+/− SD). This high value is somewhat worse relative to other mammalian microbiota studies, but this is likely due to the high abundance of reads from family S24-7, which, as mentioned, does not have a clearly resolved phylogeny^26–28^. The nickel cohort, for example, has a much lower NSTI (0.079+/− 0.036), and very low abundance of S24-7. Removing the S24-7 reads in all day 5 samples causes the NSTI to drop to 0.104+/− 0.033. To identify genes and gene pathways that significantly differentiate post-exposure control from post-exposure treated samples, we used Linear Discriminant Analysis of Effect Size (LEfSe)^29^. This program first identifies differentially abundant pathways and then performs a linear discriminant analysis to rank the pathways by their ability to discriminate two or more groups (in the case of our analysis, post-exposure samples and controls). Out of 6,909 KEGG orthologs, 926 were found to be significantly altered post-exposure, meeting the default criteria of LEfSe (a Kruskal-Wallis p-value < 0.05, and linear discriminant analysis score of ≥2). Many of these were metal specific, as shown in Supplementary Fig. S2. Significantly different KEGG pathways are shown as cladograms in Supplementary Fig. S3.

## Discussion

The knowledge of the importance of the microbiome to human health has increased in recent years, yet the role that the microbiome plays in toxic response has not fully been elucidated. It is well known that microbial metabolism of xenobiotics can affect the toxicity of different compounds^9^, but previous efforts in this area have not considered how the dynamic response and changes of the microbiota to the toxicant may alter the metabolic potential of the host microbiome. Further, these toxicant-induced changes to the microbiome may themselves induce host effects independently from the toxicant. In an effort to better understand the role of the microbiome in response to metal exposures, we have undertaken a study to examine the compositional changes of the fecal microbiota of metal-exposed mice using 16S-rRNA gene analysis. We then utilized bioinformatics techniques to infer metabolic changes in response to these exposures.

We have shown that exposure to nickel, arsenic, or cadmium by oral gavage significantly alters gut microbiota composition, while chromium and cobalt at the tested doses have only modest effects. The nickel, arsenic, and cadmium treatments showed a dose dependent effect on microbiota diversity. This suggests that the exposures occurred within a range of doses where the microbiota is sensitive and responding to the specific metal and that the changes we observed were not due to a generalized stress response in the rats. Interestingly, despite the lack of strong effects on the microbiota composition due to cobalt or chromium exposures, these exposures resulted in decreased weight and in accumulation of metals in the liver and kidney (Madejczyk *et al*., manuscript in preparation). These metals therefore had a physiological effect on the animals, but only a modest effect on the microbiota.

It should also be noted that rats were housed 2-3 animals per cage, and each cage received the same treatment. This design introduces potential cage effects, which can confound microbiota analyses^30^. However, since two cages were used per treatment, similar biases would likely have to be present in both cages to produce the significant differences we see. This is unlikely given that cage effects are generally thought to be random^31^.

The gut microbiota varied even among the inbred and co-housed rats that served as controls in this study (See Figs 4 and 5). This is unsurprising given that inter-individual variation is commonly reported to be high in microbiota surveys^32–34^. While the arsenic, cadmium, and cobalt control samples cluster together, the chromium controls form their own cluster adjacent to these three metals, and the nickel controls are well separated from all other controls (see Supplementary Fig. S4 showing just the control exposures). Despite this initial variation between cohorts, the variation within cohorts after five days of sham exposure was relatively constant (see Figs 4 and 5 and Supplementary Fig. S4), suggesting the microbiota was stable over the course of the experiment. The cohort of rats used for the nickel exposures were purchased from the same vendor as the other studies but originated from a different colony at that vendor’s facility, which likely explains the differences from the other cohorts. Fortunately, cohort-matched controls were included with each metal exposure, so the statistical comparison included animals originating from a single colony. Thus, colony source is unlikely to affect the significant differences we observed. Our findings highlight the breadth of variation in individual microbiota, which can complicate the search for signatures of exposure. For example, the phylum Verrucomicrobia increased in response to cadmium exposure (Fig. 2), but was already present in the pre-exposure nickel cohort at a comparable amount. Tracking relative changes in taxa abundance may be required for any effort to identify signatures of metal exposure based purely on compositional analyses, as the initial composition of real world samples of interest are bound to be highly variable.

As in previous studies, we found that exposure to metal compounds significantly altered the gut microbiota composition (Fig. 5, Table 1). However, the specific changes and trends reported are not always similar. Guo *et al*. reported an increase in Firmicutes and Proteobacteria, and a decrease in Bacteroidetes after arsenic exposure, which is consistent with changes we observed in the two lowest doses (Fig. 2)^20^. Our highest dose also showed an increase in Proteobacteria, but not Firmicutes or Bacteroidetes. Guo *et al*. also report a decrease in TM7, however, this phylum was prevalent in their control population but nearly absent in our own controls^20^. Breton *et al*. found that cadmium increased Lachnospiraceae abundance in mice^11^, but we saw no difference in our own study. Liu observed that Bacteroidetes increased in abundance relative to Firmicutes after cadmium exposure^17^, which, again, we did not find. While it is encouraging that some of the trends we observed were also observed by others, it is also clear that there are many discrepancies.

Metal exposure has been shown, by this paper and others, to alter gut microbiota compositions, but the specific taxa affected are not consistent. There are many potential sources of variability that could explain these discrepancies, including variation in the starting microbiota, exposure regimen (drinking water, oral gavage, etc.), time-frame of exposure, dose effects, feed composition, and technical differences in the processing and analysis of samples. We sought to limit these sources of variability as much as possible. For example, the feed used has a similar macronutrient breakdown to feed used in other studies, and is unlikely to alter the microbiome (see methods). A distinctive feature of our work is the use of a range of doses, and measuring their effects relatively early in the exposure. In addition, we used oral gavage, instead of spiking the food or water, to control the dose each animal received. We are therefore more likely to identify the changes most sensitive to metal exposure. Another serious consideration is the strength of compositional analysis when looking at higher level taxa to elucidating signatures of exposure. The sensitivity or resistance of an individual microbe to a given toxicant is determined at the gene or functional level. Species and even strain level differences may be due to genetic changes that alter sensitivity to a given toxicant. Therefore, we sought to identify functional changes within the microbiota using bioinformatics analysis to infer gene composition.

**Figure 7:**
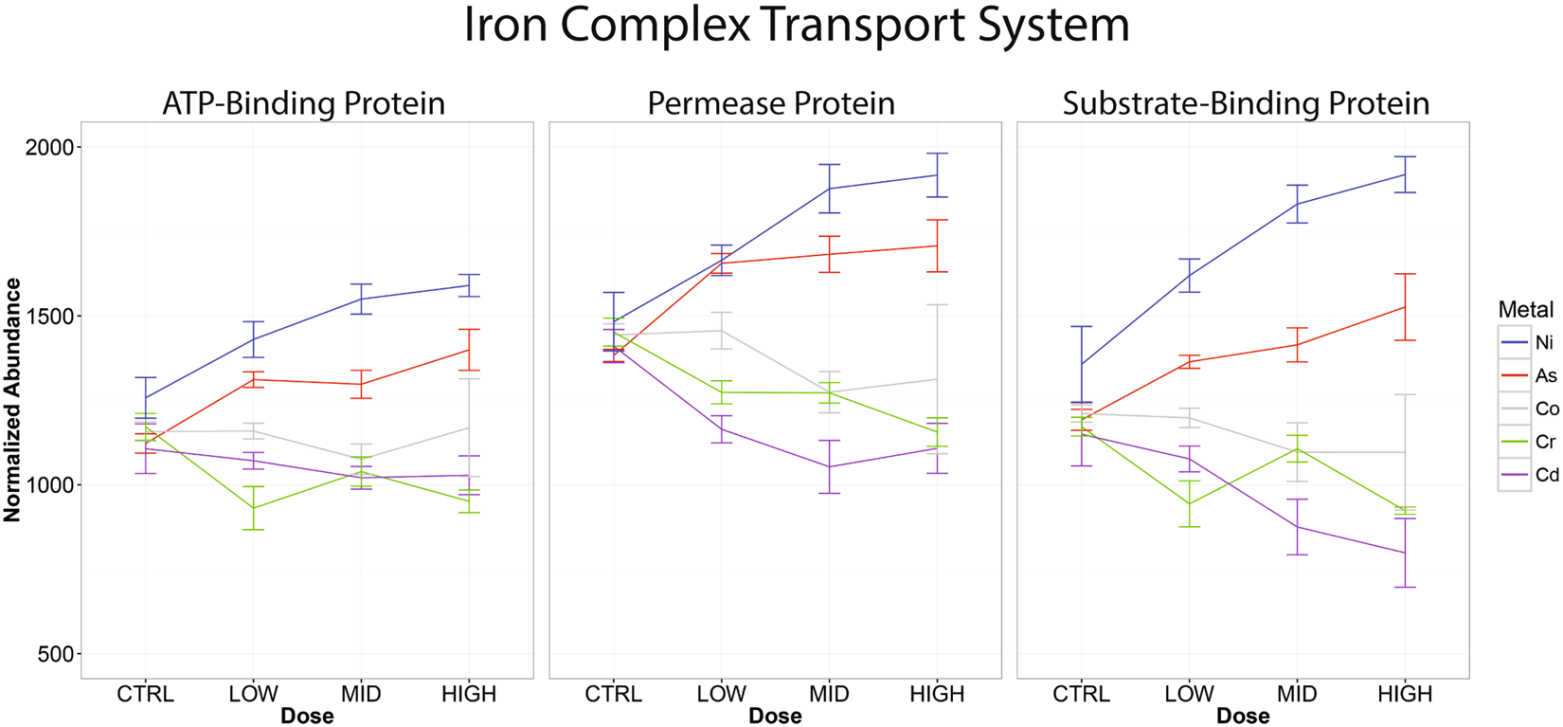
Relative abundance (+/− SEM) of KEGG orthologs belonging to the iron complex transport system, as estimated by PICRUSt.

The changes in microbial composition that we observed after metal exposure are likely related to the functional capabilities of the microbes that effect sensitivity to the toxicant or response to changes in the host. While we cannot directly measure these capabilities, we can infer them based on genomic sequence. The genes and biological pathways that were differentially abundant due to metal exposure potentially represent important mechanisms in microbial responses to the metal. One example that we observed was an increased presence of orthologs to the three genes that encode the bacterial, iron ABC-transporter system. Specifically, genes encoding the permease, ATP-binding protein, and the siderophore-binding protein (KEGG orthology groups K02013, K02015, and K02016) were significantly overrepresented in the arsenic and nickel treated samples. Figure 7 shows the relative proportion of each of these KEGG orthology groups across metal treatments.

Bacteria containing the iron complex transport system may have increased in relative abundance due to the system’s capacity to interact with metals generally. Bacterial iron importing mechanisms often can import nickel as well^35^. Siderophores have been found to also bind nickel, alleviating toxicity due to excess nickel exposure^36^. Further, iron has been observed to mitigate the effects of arsenic toxicity, so bacteria with orthologs to these iron-harvesting genes may have an advantage when growing in the presence of arsenic^20^.

Interestingly, even though the overall composition of the nickel and arsenic control samples were very different, the relative abundance of the genes was similar. The genes increased in frequency in response to higher doses, and this increase was not due to the relative increase of a particular taxa shared by the arsenic and nickel groups. Supplementary Fig. S5 shows the contribution of different taxa to the abundance of the siderophore-binding protein (KEGG ortholog K02016) in the high dose arsenic and nickel samples. Similar results were seen for the other two orthologs in this complex. The Proteobacteria phylum, and specifically the Enterobactericeae family, increased in response to both arsenic and nickel treatments. However, while Enterobacteriaceae was a major contributor to the prevalence of the iron complex transport system in nickel treated samples, it was only present in three of the arsenic treated samples (see Supplementary Fig. S5). Instead, the family S24-7 and Ruminococcaceae were the main contributors but were virtually absent from the nickel treated samples.

These finding shows the importance of characterizing the functional capacity of microbiota in addition to identifying changes in taxonomic composition. While different taxa changed in abundance due to arsenic and nickel exposure (Fig. 6), the functional analysis indicates a common set of orthologous genes increased in both sets of samples. Exposure to these metals likely created a niche where bacteria with increased repertoires of genes that mitigate metal toxicity thrived. The taxa providing these genes differed between treatments, presumably reflecting the different taxa present in the cohorts pre-exposure. This may also explain different responses to metals observed by others. The specific taxa we observe changing in response to metals may differ from other reports, but could be filling similar functional niches. Better characterization of the functional capacity of the microbiotas, for example through whole genome sequencing and metabolomics, will be required to test this idea further.

In summary, we have shown that dose dependent alteration in the microbiota can be observed and measured after exposure to the heavy metals arsenic, cadmium and nickel. This sets the ground work for the use of changes in bacterial composition as a potential biomarker of exposure. However, it must be noted, that compositional changes at higher taxonomic levels (e.g. phylum) are unlikely to be specific, as indicated by the different responses observed for the same metal across different studies^11,17,20^. By drilling down to the functional level of the gene and gene pathway, metal-specific adaptions of the microbiota can be observed. In this work, we identified a role for siderophore-importing orthologs in response to nickel and arsenic exposure, but the bacterial composition producing this response was different for each metal. As these adaptive responses are better characterized, the metabolic effects of the microbiota on toxicity and the direct effects of the microbiome on health can be better elucidated. The microbiota shows promise as a biomarker of exposure, but future work will be required to identify the functional and metabolic components most useful as a biomarker.

## Methods

### Metal Exposures/Fecal collection

Research was conducted in compliance with the Animal Welfare Act, and other Federal statutes and regulations relating to animals and experiments involving animals and adheres to principles stated in the Guide for the Care and Use of Laboratory Animals (NRC 2011) in facilities that are fully accredited by the Association for Assessment and Accreditation of Laboratory Animal Care, International. Animal studies were conducted by Integrated Laboratory Service, Inc. (ILS; Research Triangle Park, NC). All animal procedures used during the study were approved by the ILS animal care and use committee and reviewed by a Department of Defense veterinarian (ILS IACUC protocol numbers AUP 2013–14, AUP 2013–16, AUP 2013–17, AUP 2013–18, and AUP 2013–19).

CD^®^ IGS [CRL:CD (SD)] rats were purchased from Charles River Laboratories (Raleigh, NC). This strain of Sprague Dawley rats, a commonly used outbred rat model, was bred using the Charles River International Genetic Standardization (IGS) Program to ensure the same relative level of genetic heterogeneity is maintained across all breeding colonies. It has been Caesarean rederived twice: once in 1955 from the original Charles River Sprague Dawley and once in 1997 to establish the IGS isolator foundation colony. Rats were provided Purina Rodent Diet No. 5002 (Ralston Purina Co., St. Louis, MO), a standard rodent diet (13.1% calories from fat, 24.1% calories from protein, and 62.7% calories from carbohydrates), *ad libitum*. Rats had access to reverse-osmosis-treated tap water (City of Durham, NC) *ad libitum*. A total of twenty animals were used for each metal exposure. Five animals were assigned to one of three experimental groups, and five were assigned to a control group. Rats were housed with two or three animals per cage, and all cages were assigned animals from the same experimental group. Rats were exposed by oral gavage (5 mL/kg) to sodium arsenite (NaAsO_2_–15, 22, or 31 mg/kg/day), cadmium chloride (CdCl_2_ – 35, 54, or 85 mg/kg/day), sodium dichromate (Na_2_Cr_2_O_7_ – 44, 62, or 88 mg/kg/day), cobalt chloride (CoCl_2_ – 27, 47, or 82 mg/kg/day), or nickel chloride (NiCl_2_ – 177, 232, or 300 mg/kg/day) or vehicle control (water only) daily for five consecutive days. Fresh fecal samples were collected prior to the initial dosing and 24 hours after the final dosing, flash frozen, and stored at −80 °C until processing.

### Sequencing

DNA extraction, PCR amplification and amplicon preparation were performed as described in Caporaso *et al*.^37^. In summary, total DNA was extracted, and the V4 region of the 16S rRNA genes was amplified using the universal bacterial/archael primers 515 f and 806r. Three replicate PCRs were performed for each sample and then pooled. All barcoded amplicons were then pooled in equal concentrations for sequencing on the Illumina Miseq platform (Miseq Software v2.5).

### 16S rRNA amplicon Data Processing

The quality of 16S rRNA amplicon sequences was first checked using FASTQC^38^. Sequences were quality filtered and clustered into operational taxonomic units (OTUs) following the standard QIIME analysis framework^39^. First, paired-end reads were joined together with the join_paired_ends.py script from QIIME, using the fastq-join tool and the default settings^40^. Chimeric read identification and removal was done using usearch6.1^41^. Reads were clustered into OTUs using the pick_open_reference.py script from QIIME, which implements both reference based (using the Greengenes v13.8 97% reference OTU database^42^), as well as *de novo* OTU clustering. The default options were used. OTUs were clustered at 97% sequence similarity. The Uclust algorithm was used for both OTU clustering and taxonomic assignment to the Greengenes database^41^. OTUs with less than 2 sequences were eliminated. PyNAST was used to align representative OTU sequences for tree construction, and any OTU sequences with a sequence identity match of less than 75% were eliminated^39,43^. Samples were excluded if they had fewer than 3,544 reads (which is equal to the experiment-wide mean minus 2 times the experiment-wide standard deviation) or were blanks or technical controls. The mean reads per sample was 15,317.

### Visualization of Taxonomic Distribution

Stacked bar plots showing taxonomic summaries were based on percent abundance and averaged across all samples in that group (Fig. 2). Archaea, though present at very low levels, were excluded from summaries. Only phyla with an abundance of at least 5% in one of the samples within the cohort are indicated by their own bar. Phyla not meeting these criteria are collapsed into the “Other” category. Reads that could not be assigned to any taxa were placed in the “Unassigned” category. At levels below phylum, (Fig. 2, middle and bottom rows) taxa that made up more than 5% of the total reads within a sample are indicated by their own bar shaded with a tone matching the color of its phylum. All bar plots were generated in R, version 3.3.0^44^.

### Statistical analysis

Statistical analysis of beta and alpha diversity were performed in R (version 3.3.0), using the “phyloseq” package^45^. Alpha diversity measures were calculated using the “estimate_richness” function for OTU richness and Shannon’s Diversity, and Faith’s phylogenetic distance was calculated using the “pd” function of the “picante” R package^46^. One-way ANOVA was used to calculate if dose significantly altered alpha diversity. The Kruskal-Wallis test was used to test for significant changes to alpha diversity, and the Holm method was used to correct for multiple comparisons. Principal Coordinate Analyses were performed using the “ordinate” function. The PERMANOVA and homogeneity of variance tests were run with the “adonis” and “betadisper” functions, respectively, from the “vegan” package^47^. Pairwise PERMANOVA tests were conducted between post-exposure treated samples and either pre-exposure samples, or post-exposure control samples to test significance of treatment effect. Significant dose effects were calculated using the adonis function, specifying the treatment levels (control, low, mid, and high) as Helmert contrasts. The PERMANOVA test is a non-parametric, multivariate analysis of variance method that can be applied to a distance matrix. This test assumes equality of variance between the groups being tested, so a multivariate homogeneity of variance test, using the “betadisper” function in the vegan R package was performed for each PERMANOVA test, using the same data and comparisons. For each PERMANOVA test, the PERMANOVA *p*-value and the effect size (proportion of variance explained by the grouping variable), and the betadisper variance test *p*-value are reported in Table 1. Differential abundance analysis was conducted using the DESeq. 2 package in R, in conjunction with the phyloseq package^48,49^. The Wald test was used to test for a significant association between taxa abundance and exposure status within each cohort.

### Biological Inferences

Estimations of gene and biological pathway effects were performed using PICRUSt^26^. Only OTUs matching the Greengenes database (not those identified by de novo clustering) were used as input for PICRUSt, following the guidance of the authors. The nearest sequenced taxon index (NSTI) was calculated for each day 5 (post-treatment) sample, as a measure of how well microbes in the sample are represented by the sequenced genomes used to make gene content inferences. OTU abundances were normalized based on 16S copy number, and samples were rarefied to the depth of the sample with the fewest reads (2987) using single_rarefaction.py in QIIME. Gene abundances were calculated using predict_metagenomes.py, the main PICRUSt script. KEGG pathway abundances were calculated based on gene abundances using the categorize_by_function.py script, a PICRUSt utility script. Genes and gene pathways contributing to the difference between groups were identified using the Linear Discriminant Analysis of Effect Size (LEfSe) program and default settings^29^.

### Availability of data and materials

The dataset generated and analyzed during this study is submitted to the European Nucleotide Archive, and will be made available on publication.

### Disclaimer

Opinions, interpretations, conclusions, and recommendations are those of the author and are not necessarily endorsed by the U.S. Army. Citations of commercial organizations or trade names in this report do not constitute an official Department of the Army endorsement or approval of the products or services of these organizations.

## Electronic supplementary material

Supplemental Information
