# Supplementary material for: Exposure to toxic metals triggers unique responses from the rat gut microbiota
Source: Sci Rep. 2018 Apr 26;8:6578. doi: 10.1038/s41598-018-24931-w (PMC5919903; doi:10.1038/s41598-018-24931-w)
Supplement: Supplementary file 1 — Supplemental Information [file 41598_2018_24931_MOESM1_ESM.pdf]

Supplementary Information for:

Exposure to toxic metals triggers unique responses from the rat gut microbiota

Joshua B. Richardson, Blair C. R. Dancy, Cassandra L. Horton, Young S. Lee, Michael S. Madejczyk, Zhenjiang Zech Xu Gail Ackermann, Gregory Humphrey, Gustavo Palacios, Rob Knight, John A. Lewis

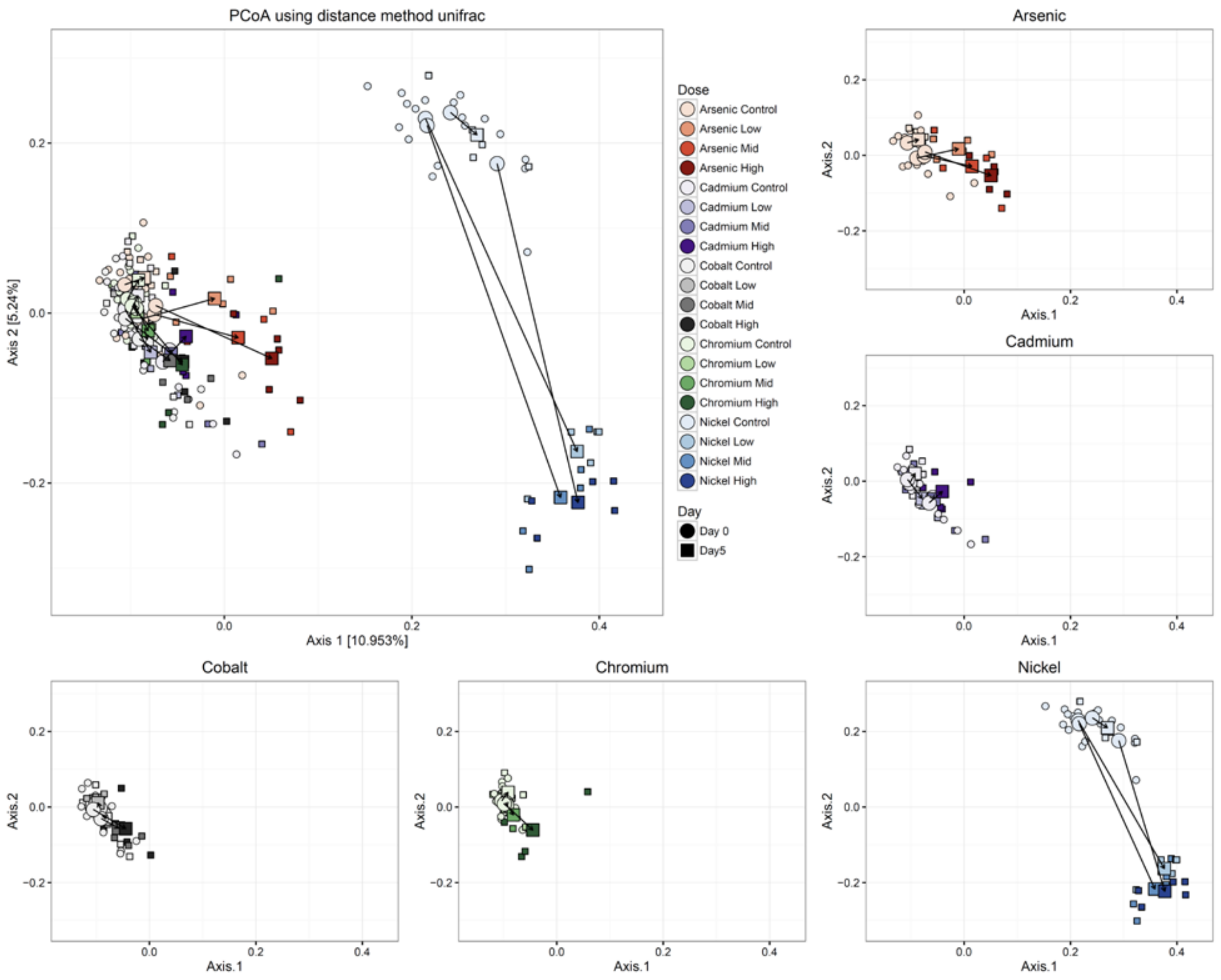

Figure S1. Principal Coordinate Analysis using the UniFrac distance metric. The main figure shows first two coordinate axes from a Principal Coordinate Analysis based on the UniFrac distance. Shape indicates day of sampling. Color indicates type of metal exposure, and shading indicates dose level. The small shapes indicate individual samples, and large shapes indicate centroids. Arrows connect the pre- and post-exposure centroids for each cohort.

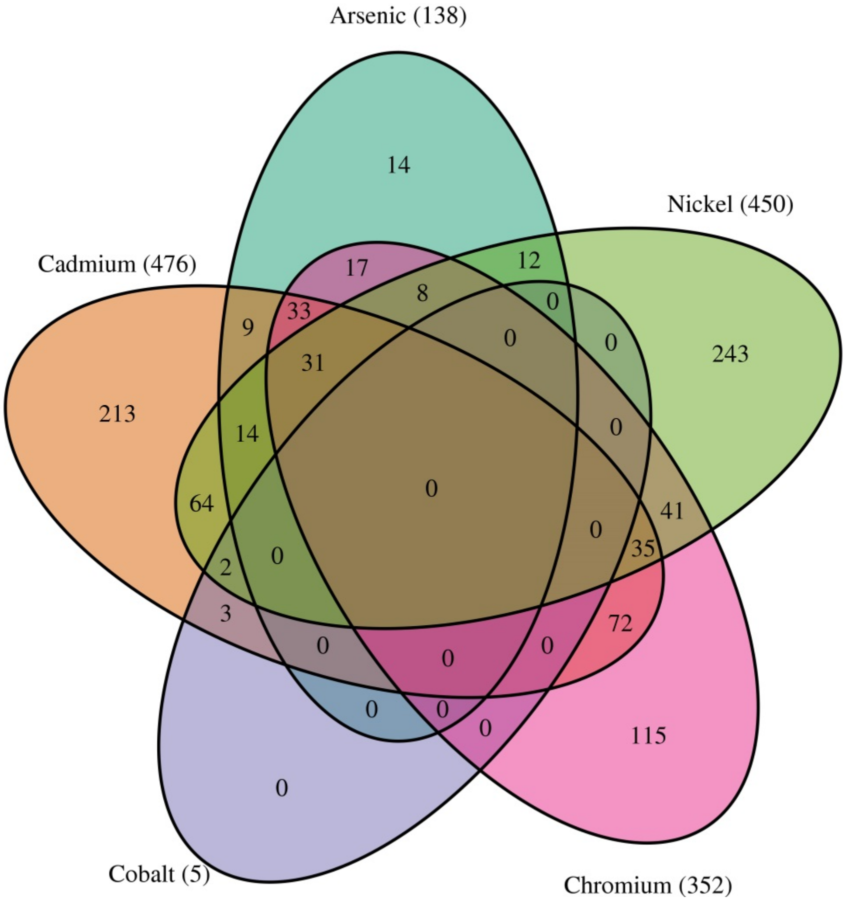

Figure S2. Venn diagram showing number of KEGG orthologs significantly different according to LEfSe (Kruskal Wallis test <0.05 and LDA score >2), for each metal.

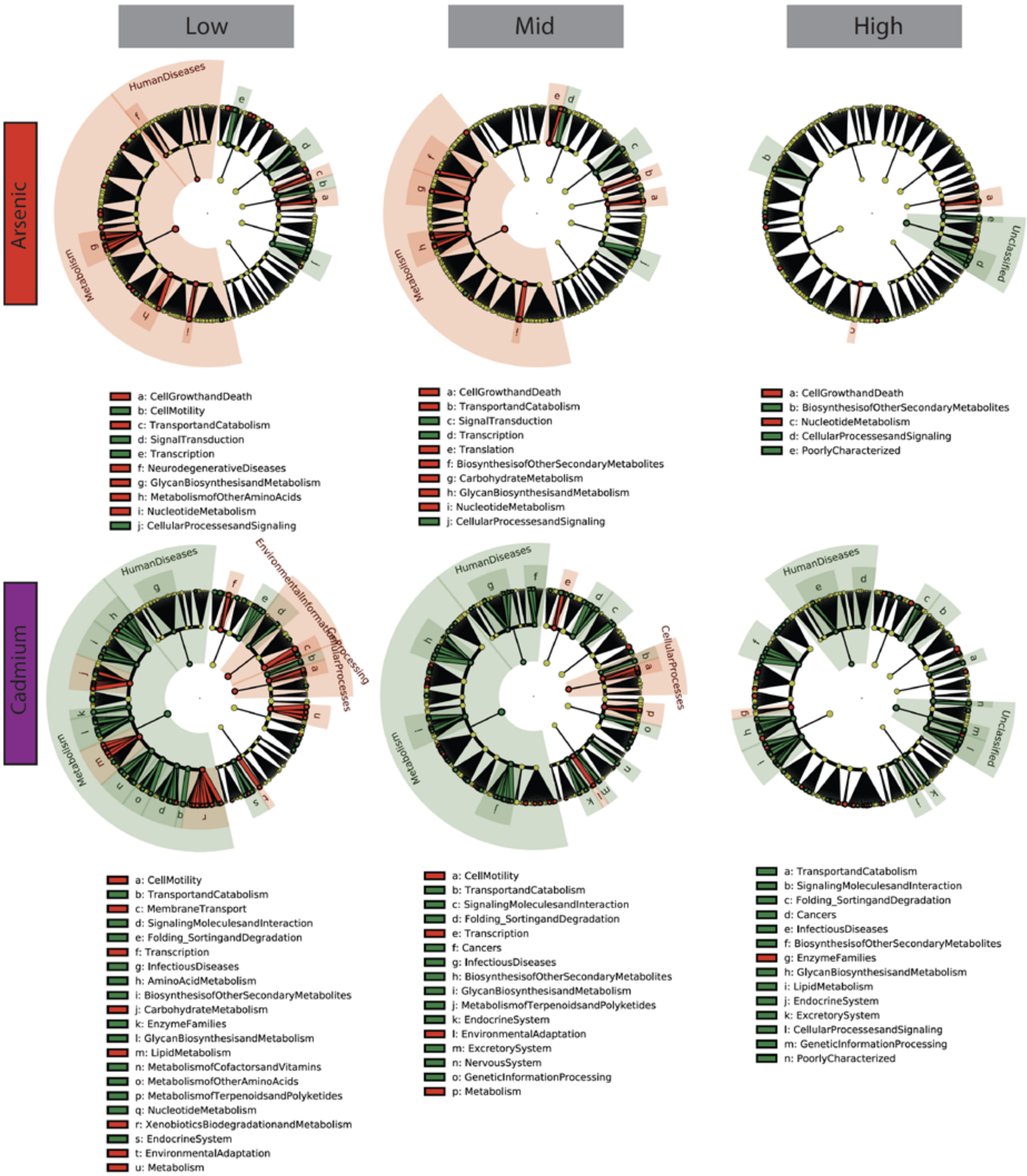
Low
Mid
High
Arsenic
Cadmium
HumanDiseases
Metabolism
Unclassified
EnvironmentalInformationProcessing
CellularProcesses
a: CellGrowthandDeath
b: CellMotility
c: TransportandCatabolism
d: SignalTransduction
e: Transcription
f: NeurodegenerativeDiseases
g: GlycanBiosynthesisandMetabolism
h: MetabolismofOtherAminoAcids
i: NucleotideMetabolism
j: CellularProcessesandSignaling
a: CellGrowthandDeath
b: TransportandCatabolism
c: SignalTransduction
d: Transcription
e: Translation
f: BiosynthesisofOtherSecondaryMetabolites
g: CarbohydrateMetabolism
h: GlycanBiosynthesisandMetabolism
i: NucleotideMetabolism
j: CellularProcessesandSignaling
a: CellGrowthandDeath
b: BiosynthesisofOtherSecondaryMetabolites
c: NucleotideMetabolism
d: CellularProcessesandSignaling
e: PoorlyCharacterized
a: CellMotility
b: TransportandCatabolism
c: MembraneTransport
d: SignalingMoleculesandInteraction
e: Folding_SortingandDegradation
f: Transcription
g: InfectiousDiseases
h: AminoAcidMetabolism
i: BiosynthesisofOtherSecondaryMetabolites
j: CarbohydrateMetabolism
k: EnzymeFamilies
l: GlycanBiosynthesisandMetabolism
m: LipidMetabolism
n: MetabolismofCofactorsandVitamins
o: MetabolismofOtherAminoAcids
p: MetabolismofTerpenoidsandPolyketides
q: NucleotideMetabolism
r: XenobioticsBiodegradationandMetabolism
s: EndocrineSystem
t: EnvironmentalAdaptation
u: Metabolism
a: CellMotility
b: TransportandCatabolism
c: SignalingMoleculesandInteraction
d: Folding_SortingandDegradation
e: Transcription
f: Cancers
g: InfectiousDiseases
h: BiosynthesisofOtherSecondaryMetabolites
i: GlycanBiosynthesisandMetabolism
j: MetabolismofTerpenoidsandPolyketides
k: EndocrineSystem
l: EnvironmentalAdaptation
m: ExcretorySystem
n: NervousSystem
o: GeneticInformationProcessing
p: Metabolism
a: TransportandCatabolism
b: SignalingMoleculesandInteraction
c: Folding_SortingandDegradation
d: Cancers
e: InfectiousDiseases
f: BiosynthesisofOtherSecondaryMetabolites
g: EnzymeFamilies
h: GlycanBiosynthesisandMetabolism
i: LipidMetabolism
j: EndocrineSystem
k: ExcretorySystem
l: CellularProcessesandSignaling
m: GeneticInformationProcessing
n: PoorlyCharacterized

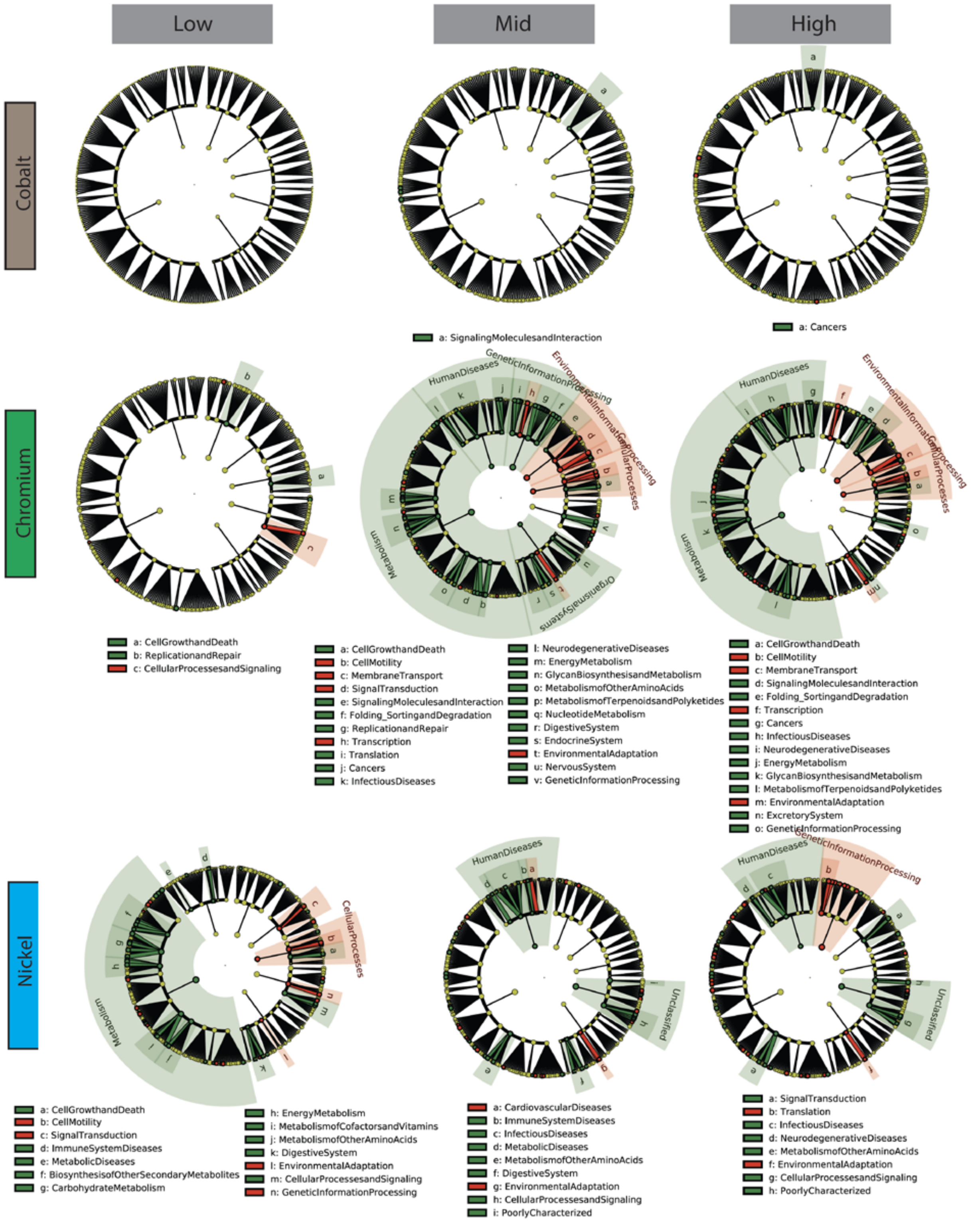

Figure S3. Cladograms representing KEGG pathways. Red nodes are significantly more abundant in the day 5 controls, according to LEfSe (Kruskal Wallis test <0.05 and LDA score >2), and green nodes are significantly more abundant in the exposed samples.

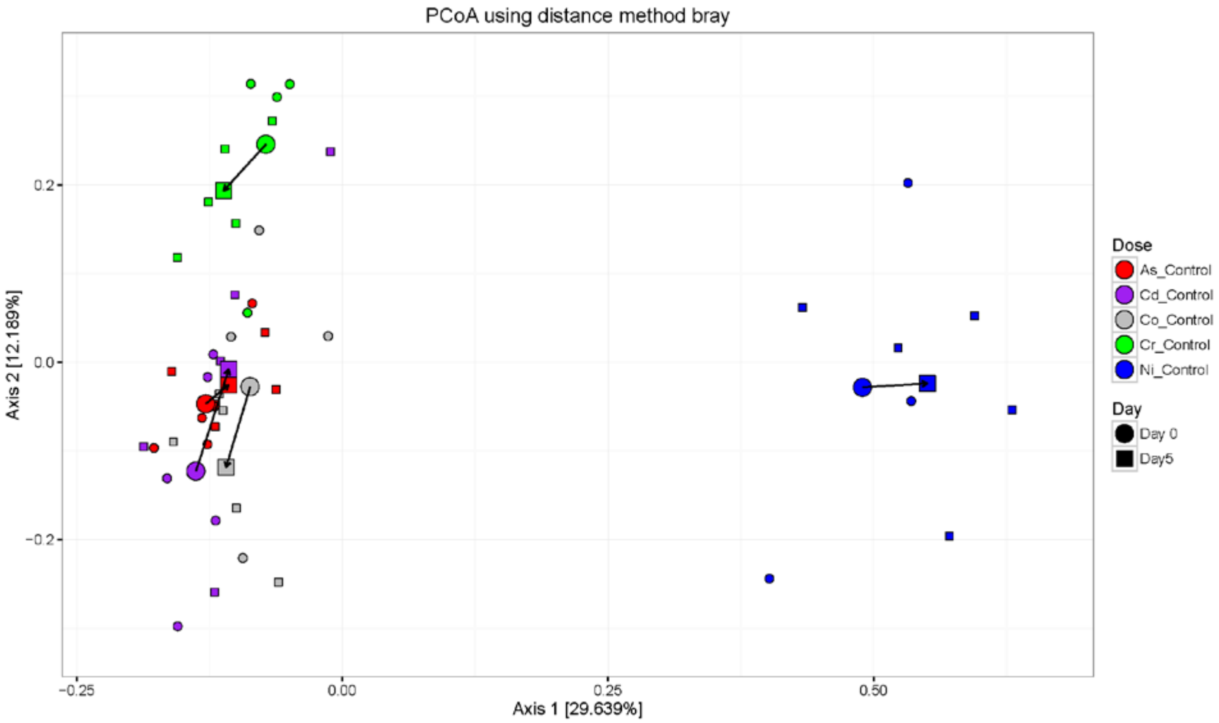

Figure S4. PCoA of sham exposures for each metal cohort. Large symbols are centroids and arrows connect pre and post-exposure samples.

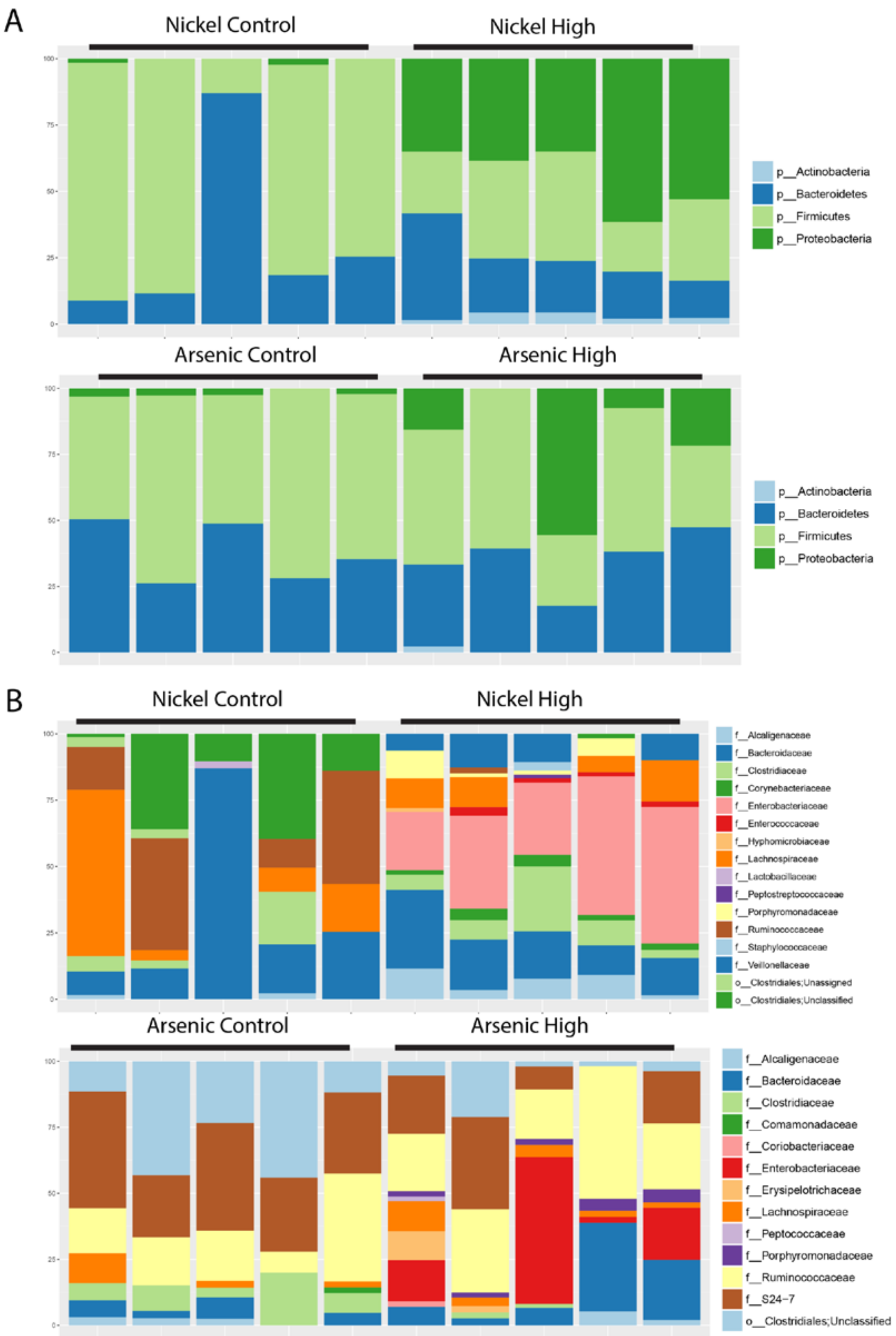

Figure S5. A. Relative contributions of phylum to abundance of KEGG orthologous gene K02016, a component of the iron transport complex. Each bar represents a single sample. B. Relative contributions of families to abundance of KEGG orthologous gene K02016.

Supplemental Table S1. Results from the DESeq analysis, showing all genera that were significantly differentially abundant in at least one comparison. Results from each comparison (for example, Nickel high vs. Nickel control) are shown separately, as indicated by the main header. The sub headers refer to the experimental group's values (for example, "Mean" indicates normalized mean of the experimental group; positive fold change values indicate higher values in the experimental group relative to the control group, etc.). "Sig." indicates the test had an adjusted p-value of 0.05 (Benjamini-Hochberg method).

#	Taxonomy
1	k__Bacteria;p__Acidobacteria;c__Acidobacteria-6;o__iii1-15;f__;g__
2	k__Bacteria;p__Actinobacteria;c__Actinobacteria;o__Actinomycetales;f__Corynebacteriaceae;g__Corynebacterium
3	k__Bacteria;p__Actinobacteria;c__Actinobacteria;o__Bifidobacteriales;f__Bifidobacteriaceae;g__Bifidobacterium
4	k__Bacteria;p__Actinobacteria;c__Coriobacteriia;o__Coriobacteriales;f__Coriobacteriaceae;g__Adlercreutzia
5	k__Bacteria;p__Bacteroidetes;c__Bacteroidia;o__Bacteroidales;f__[Odoribacteraceae];g__Odoribacter
6	k__Bacteria;p__Bacteroidetes;c__Bacteroidia;o__Bacteroidales;f__Bacteroidaceae;g__Bacteroides
7	k__Bacteria;p__Bacteroidetes;c__Bacteroidia;o__Bacteroidales;f__Porphyromonadaceae;g__Parabacteroides
8	k__Bacteria;p__Bacteroidetes;c__Bacteroidia;o__Bacteroidales;f__S24-7;g__
9	k__Bacteria;p__Cyanobacteria;c__4C0d-2;o__YS2;f__;g__
10	k__Bacteria;p__Deferribacteres;c__Deferribacteres;o__Deferribacterales;f__Deferribacteraceae;g__Mucispirillum
11	k__Bacteria;p__Firmicutes;c__Bacilli;o__Bacillales;f__Staphylococcaceae;g__Staphylococcus
12	k__Bacteria;p__Firmicutes;c__Bacilli;o__Lactobacillales;f__Aerococcaceae;g__Aerococcus
13	k__Bacteria;p__Firmicutes;c__Bacilli;o__Lactobacillales;f__Aerococcaceae;g__Facklamia
14	k__Bacteria;p__Firmicutes;c__Bacilli;o__Lactobacillales;f__Enterococcaceae;g__
15	k__Bacteria;p__Firmicutes;c__Bacilli;o__Lactobacillales;f__Enterococcaceae;g__Enterococcus
16	k__Bacteria;p__Firmicutes;c__Bacilli;o__Lactobacillales;f__Lactobacillaceae;g__Lactobacillus
17	k__Bacteria;p__Firmicutes;c__Bacilli;o__Lactobacillales;f__Streptococcaceae;g__Lactococcus
18	k__Bacteria;p__Firmicutes;c__Bacilli;o__Lactobacillales;f__Streptococcaceae;g__Streptococcus
19	k__Bacteria;p__Firmicutes;c__Bacilli;o__Turicibacterales;f__Turicibacteraceae;g__Turicibacter
20	k__Bacteria;p__Firmicutes;c__Clostridia;o__Clostridiales;f__;g__
21	k__Bacteria;p__Firmicutes;c__Clostridia;o__Clostridiales;f__[Mogibacteriaceae];g__
22	k__Bacteria;p__Firmicutes;c__Clostridia;o__Clostridiales;f__Clostridiaceae;g__
23	k__Bacteria;p__Firmicutes;c__Clostridia;o__Clostridiales;f__Clostridiaceae;g__Clostridium
24	k__Bacteria;p__Firmicutes;c__Clostridia;o__Clostridiales;f__Dehalobacteriaceae;g__Dehalobacterium
25	k__Bacteria;p__Firmicutes;c__Clostridia;o__Clostridiales;f__Lachnospiraceae;g__
26	k__Bacteria;p__Firmicutes;c__Clostridia;o__Clostridiales;f__Lachnospiraceae;g__[Ruminococcus]
27	k__Bacteria;p__Firmicutes;c__Clostridia;o__Clostridiales;f__Lachnospiraceae;g__Blautia
28	k__Bacteria;p__Firmicutes;c__Clostridia;o__Clostridiales;f__Lachnospiraceae;g__Dorea
29	k__Bacteria;p__Firmicutes;c__Clostridia;o__Clostridiales;f__Lachnospiraceae;g__Epulopiscium
30	k__Bacteria;p__Firmicutes;c__Clostridia;o__Clostridiales;f__Lachnospiraceae;g__Roseburia
31	k__Bacteria;p__Firmicutes;c__Clostridia;o__Clostridiales;f__Peptococcaceae;g__
32	k__Bacteria;p__Firmicutes;c__Clostridia;o__Clostridiales;f__Peptococcaceae;g__rc4-4
33	k__Bacteria;p__Firmicutes;c__Clostridia;o__Clostridiales;f__Ruminococcaceae;g__
34	k__Bacteria;p__Firmicutes;c__Clostridia;o__Clostridiales;f__Ruminococcaceae;g__Oscillospira
35	k__Bacteria;p__Firmicutes;c__Clostridia;o__Clostridiales;f__Ruminococcaceae;g__Ruminococcus
36	k__Bacteria;p__Firmicutes;c__Clostridia;o__Clostridiales;f__Veillonellaceae;g__Veillonella
37	k__Bacteria;p__Firmicutes;c__Erysipelotrichi;o__Erysipelotrichales;f__Erysipelotrichaceae;g__Allobaculum
38	k__Bacteria;p__Proteobacteria;c__Alphaproteobacteria;o__RF32;f__;g__
39	k__Bacteria;p__Proteobacteria;c__Alphaproteobacteria;o__Rhizobiales;f__Rhodobiaceae;g__Afifella
40	k__Bacteria;p__Proteobacteria;c__Alphaproteobacteria;o__Sphingomonadales;f__Erythrobacteraceae;g__
41	k__Bacteria;p__Proteobacteria;c__Betaproteobacteria;o__Burkholderiales;f__Alcaligenaceae;g__Sutterella
42	k__Bacteria;p__Proteobacteria;c__Gammaproteobacteria;o__Enterobacteriales;f__Enterobacteriaceae;g__
43	k__Bacteria;p__Proteobacteria;c__Gammaproteobacteria;o__Enterobacteriales;f__Enterobacteriaceae;g__Escherichia
44	k__Bacteria;p__Proteobacteria;c__Gammaproteobacteria;o__Enterobacteriales;f__Enterobacteriaceae;g__Morganella
45	k__Bacteria;p__Proteobacteria;c__Gammaproteobacteria;o__Enterobacteriales;f__Enterobacteriaceae;g__Proteus
46	k__Bacteria;p__Tenericutes;c__Mollicutes;o__RF39;f__;g__
47	k__Bacteria;p__Verrucomicrobia;c__Verrucomicrobiae;o__Verrucomicrobiales;f__Verrucomicrobiaceae;g__Akkermansia

	Nickel High						
#	Mean	Fold Change	Fold Change SE	Wald Statistic	P value	Adjusted P value	Significant
1	4.497276442	-1.715175859	1.723465964	-0.995189864	0.31964393	0.546916414	
2	85.87432949	5.191205801	1.087494154	4.773548235	1.81E-06	1.66E-05	*
3	195.1644025	5.120219241	1.179103254	4.342468925	1.41E-05	0.000101642	*
4	2.330339722	-4.087103247	1.58658551	-2.576037171	0.00999399	0.034590402	*
5	1.852584245	-3.727556528	1.777910251	-2.096594317	0.036029503	0.101082772	
6	3387.446511	0.635458658	0.954466827	0.665773435	0.505555927	0.744396287	
7	549.4981822	3.517914321	1.029331821	3.417667897	0.000631601	0.003189586	*
8	2936.053886	-8.913640701	0.998037975	-8.931163864	4.22E-19	2.13E-17	*
9	7.101339543	-4.253966734	1.455623781	-2.922435583	0.003473055	0.012991797	*
10	4.608580673	-3.567086478	1.69941638	-2.099006765	0.035816304	0.101082772	
11	57.78772249	5.300192522	1.22103882	4.340724009	NA	NA	
12	4.59715001	5.026866257	1.631606563	3.080930398	0.002063549	0.008163541	*
13	6.710146004	5.632472921	1.5045582	3.743605877	0.000181398	0.001221414	*
14	6.063728857	5.361419351	1.648187108	3.252919116	0.00114226	0.005244012	*
15	192.8914703	7.273831617	1.072035673	6.785064899	1.16E-11	1.95E-10	*
16	978.1177198	-0.411488534	0.857669048	-0.479775427	0.631387088	0.821415902	
17	7.881236071	4.725329234	1.316151064	3.590263582	0.000330344	0.001957308	*
18	8.380583338	0.940130257	1.211519657	0.775992573	0.437753371	0.66989531	
19	361.9956316	-0.02243384	1.195719417	-0.018761793	0.985031133	0.999631281	
20	1524.616253	-4.421342327	0.969855156	-4.558765606	5.15E-06	4.33E-05	*
21	6.862219537	-1.631492761	1.327565105	-1.228936159	0.219095743	0.388222281	
22	1067.906392	2.22846918	0.953001118	2.338369955	0.019368065	0.059278017	
23	48.62162342	0.230340769	1.393091195	0.165345076	0.868672382	0.927172775	
24	2.362053016	-4.089628321	1.622880341	-2.519981429	0.011736103	0.038236979	*
25	271.870764	-1.444817632	0.885985396	-1.630746556	0.102943809	0.234285656	
26	142.9318399	4.135090586	0.862418223	4.794762535	1.63E-06	1.64E-05	*
27	208.1272181	-0.551859207	1.056478426	-0.522357289	0.601421588	0.805682146	
28	120.0602466	3.242501295	0.912647747	3.552850819	0.000381081	0.002025744	*
29	5.134845514	4.862628557	1.894686387	2.566455637	0.010274377	0.034590402	*
30	26.98296147	-4.515189672	1.581654343	-2.854725934	0.004307398	0.015537401	*
31	3.361885955	-0.705423065	1.725775476	-0.40875715	0.682717889	0.821415902	
32	20.63969504	-4.441267071	1.375831816	-3.228059578	0.00124633	0.005473015	*
33	540.8843681	-0.945821868	0.714023889	-1.324636168	0.185291854	0.347820123	
34	184.9845206	-3.414448808	1.179987324	-2.893631769	NA	NA	
35	222.1126308	-2.96874886	0.830175812	-3.576048369	0.000348827	0.001957308	*
36	418.8463208	8.148409106	1.341766853	6.072894922	1.26E-09	1.81E-08	*
37	0	NA	NA	NA	NA	NA	
38	11.72207392	-6.209852371	1.595739877	-3.891519201	NA	NA	
39	4.647494365	-2.529175884	1.55541123	-1.626049649	0.103939094	0.234285656	
40	4.05640999	4.585663821	1.882098453	2.436463307	0.014831674	0.046812472	*
41	723.1914339	3.531054915	0.963771889	3.663786997	0.000248513	0.001568741	*
42	1099.259169	8.430236475	1.198354327	7.034844609	1.99E-12	4.03E-11	*
43	3.766764735	4.870087929	1.51877114	3.206597625	0.001343147	0.005652411	*
44	126.4803249	9.671773505	1.341545226	7.209427844	5.62E-13	1.42E-11	*
45	218.7904542	6.831335395	1.153912499	5.920150274	3.22E-09	3.61E-08	*
46	94.90911965	-6.792125691	1.126431603	-6.029771955	1.64E-09	2.07E-08	*
47	466.7761735	-9.556836998	1.320227114	-7.238782553	4.53E-13	1.42E-11	*

	Nickel Mid						
#	Mean	Fold Change	Fold Change SE	Wald Statistic	P value	Adjusted P value	Significant
1	4.565567876	-1.955458945	1.674644978	-1.167685671	0.242933579	0.346039129	
2	63.08745365	4.626654932	1.414137196	3.271715747	0.00106897	0.005847894	*
3	157.825234	4.76011793	1.25698132	3.786944049	0.000152511	0.001289415	*
4	2.367448467	-3.565268319	1.557296124	-2.289396514	0.022056324	0.066168973	
5	1.682903698	-3.559975545	1.801242027	-1.976400446	0.048109441	0.12092373	
6	4306.722324	1.067362201	1.019488138	1.046958922	NA	NA	
7	1003.715319	4.354834844	1.024443885	4.250925706	2.13E-05	0.000282838	*
8	3071.579319	-4.671292357	1.213055981	-3.850846483	0.00011771	0.001094706	*
9	6.368829546	-4.615695972	1.452188901	-3.17844047	0.001480696	0.007247618	*
10	4.027878657	-4.5402813	1.830973561	-2.479708826	0.013148971	0.0452909	*
11	28.39927353	4.283966352	1.262013166	3.394549652	0.000687416	0.004261978	*
12	10.97194929	6.195882018	1.604071326	3.862597578	NA	NA	
13	4.99428861	5.03114447	1.76354366	2.85286074	0.00433276	0.017519423	*
14	10.82481748	6.083392341	1.68012457	3.620798392	0.000293695	0.002137072	*
15	277.7538022	7.689274709	1.291436911	5.954045951	2.62E-09	8.11E-08	*
16	957.6832838	-0.455925183	0.95623902	-0.47678998	0.633511682	0.745779575	
17	9.279719918	4.752555	1.626207033	2.922478444	0.003472577	0.015378553	*
18	6.969666868	0.508344385	1.325010145	0.383653202	0.701235502	0.785721708	
19	377.2944451	0.473464484	1.173154386	0.403582418	0.686519817	0.778613938	
20	1807.70422	-1.449639334	0.892827899	-1.623649233	0.104450692	0.206679028	
21	6.568881095	-1.442892713	1.242628602	-1.161161678	0.245576156	0.346039129	
22	980.4808575	2.267588243	0.940362384	2.411398288	0.015891484	0.052782428	
23	20.94361126	-3.155828698	1.072558103	-2.942338218	0.003257439	0.015147093	*
24	2.257637621	-3.966407595	1.653717639	-2.398479343	0.016463305	0.052796116	
25	231.6618163	-1.937218329	0.97948406	-1.977794645	0.047951878	0.12092373	
26	71.93125586	3.201775015	0.963889773	3.321723195	0.000894634	0.00520006	*
27	194.6579575	-0.742812192	1.119301515	-0.663639049	0.506921359	0.628582485	
28	43.32380388	1.741994873	0.927643758	1.877870528	0.060398879	0.144028096	
29	0.932089344	2.552564138	2.233277731	1.142967622	NA	NA	
30	25.07155144	-5.807568456	1.476669474	-3.932883124	8.39E-05	0.000867308	*
31	2.446517484	-2.603917738	1.704734236	-1.527462571	0.126646028	0.240368991	
32	24.43053514	-2.324469	1.324787598	-1.754597494	0.07932818	0.171570251	
33	667.8325317	-0.017781726	0.917439455	-0.019381907	0.984536444	0.984536444	
34	212.3594271	-1.203518604	1.264230336	-0.951977317	NA	NA	
35	216.1634989	-2.427240876	0.960936243	-2.525912509	0.011539822	0.041277054	*
36	19.63309077	3.85311306	1.424511568	2.704866108	0.006833193	0.025419476	*
37	0	NA	NA	NA	NA	NA	
38	10.37291196	-5.442003334	1.504812999	-3.616398408	0.00029873	0.002137072	*
39	4.316737861	-4.710890245	1.715441241	-2.746168235	0.006029584	0.023364636	*
40	0.354339463	1.656680202	2.248796524	0.736696355	0.461307024	NA	
41	1163.709256	4.206693164	1.048516171	4.012044146	6.02E-05	0.00069977	*
42	867.2722779	7.658075625	1.469102383	5.212758288	1.86E-07	4.33E-06	*
43	2.217104993	3.831318902	1.920419483	1.995042716	0.046038221	0.12092373	
44	185.9394524	9.355220996	1.812514126	5.161461013	2.45E-07	4.56E-06	*
45	372.8379166	7.160387323	1.502195059	4.766616212	1.87E-06	2.90E-05	*
46	101.3784079	-3.122411186	1.081788024	-2.886342903	0.003897473	0.01647568	*
47	491.0453307	-10.51704931	1.414320641	-7.436113849	1.04E-13	9.64E-12	*

	Nickel Low						
#	Mean	Fold Change	Fold Change SE	Wald Statistic	P value	Adjusted P value	Significant
1	4.530536795	-4.659588922	1.477485458	-3.153729125	0.001611986	0.012251096	*
2	3.834524311	-1.439159121	1.465114773	-0.982284219	0.325959847	0.530848893	
3	81.14453646	3.518626	0.963068313	3.653558062	0.000258631	0.00268036	*
4	3.728687796	-2.007381481	1.345932823	-1.49144255	0.135845344	0.303654298	
5	2.961221234	-4.638240946	1.533457336	-3.024695137	0.002488839	0.016188623	*
6	3560.28996	0.31517875	0.76157008	0.413853902	0.678981105	0.803676941	
7	777.334964	3.751548583	0.775857079	4.835360385	1.33E-06	2.16E-05	*
8	3878.282021	-4.951195513	0.909883769	-5.44156922	5.28E-08	1.20E-06	*
9	10.65495571	-4.631994228	1.243130827	-3.726071406	0.000194487	0.002217156	*
10	6.417375226	-5.493415411	1.573295462	-3.491661639	0.000480026	0.004560246	*
11	12.06934597	2.333478386	1.024870234	2.276852531	0.022795033	0.099947454	
12	0	NA	NA	NA	NA	NA	
13	0.183143252	0.168709768	2.013469008	0.083790596	0.933222925	NA	
14	0.891475006	2.098702963	1.813945958	1.156982077	0.247279683	NA	
15	15.60994589	2.98797078	1.179479135	2.533296852	0.011299522	0.058552066	
16	1443.563868	-0.269450701	0.661647893	-0.407241833	0.68383038	0.803676941	
17	0.708639827	0.47438461	1.995213798	0.237761292	0.812066243	NA	
18	6.861152599	-0.436078971	1.058476109	-0.411987543	0.680348554	0.803676941	
19	522.0789892	0.165370821	1.014156109	0.16306249	0.870469233	0.936165024	
20	2137.396098	-4.810329661	0.734954558	-6.545070863	5.95E-11	3.39E-09	*
21	8.019113304	-2.918974395	1.10313153	-2.646080104	0.008143053	0.044205145	*
22	802.0317568	1.129648802	0.849606857	1.329613565	0.183645626	0.367291253	
23	34.96889614	-2.847448872	1.038099843	-2.742943168	0.006089122	0.034707997	*
24	3.333846362	-4.863595716	1.434321071	-3.3908696	0.000696712	0.006109632	*
25	430.0708151	-1.455582804	0.785734246	-1.85251287	0.063952211	0.191343191	
26	129.3532343	3.317463118	0.824259499	4.024779967	5.70E-05	0.000722362	*
27	306.1020241	-0.727572051	0.891453358	-0.816163902	0.4144064	0.60145627	
28	98.11854924	2.275398739	0.749244275	3.036925093	0.002390048	0.016188623	*
29	0	NA	NA	NA	NA	NA	
30	42.47765963	-7.596153537	1.414671728	-5.369552091	7.89E-08	1.50E-06	*
31	2.835031806	-4.492155365	1.563434292	-2.873261377	0.004062579	0.024375471	*
32	29.86298213	-1.90338693	0.933162926	-2.03971555	0.041378665	0.157238928	
33	715.335657	-1.507449497	0.631764737	-2.386093127	0.017028438	0.082277189	
34	281.0756794	-4.221379441	0.969009913	-4.356384165	NA	NA	
35	330.9639791	-3.748615144	0.804201836	-4.661286476	3.14E-06	4.48E-05	*
36	352.1256609	7.484338828	1.249767917	5.98858294	2.12E-09	6.03E-08	*
37	0	NA	NA	NA	NA	NA	
38	18.57241163	-7.128954703	1.381844382	-5.159014138	NA	NA	
39	6.424621227	-1.411475274	1.28534639	-1.098128322	0.27214846	0.463058573	
40	1.422151116	2.626902111	1.7975107	1.461411112	0.143902653	0.315478893	
41	410.0598449	2.260683259	0.749409306	3.016620214	0.002556098	0.016188623	*
42	8.227387703	0.768836869	1.182608538	0.650119498	0.515615035	0.691530753	
43	0	NA	NA	NA	NA	NA	
44	0	NA	NA	NA	NA	NA	
45	2.004799665	-3.569990192	1.563252677	-2.283693638	0.022389542	0.099947454	
46	125.0828873	-5.537341019	0.87104581	-6.357118022	2.06E-10	7.81E-09	*
47	587.35443	-10.85948326	1.148419889	-9.456021588	3.20E-21	3.65E-19	*

	Chromium High						
#	Mean	Fold Change	Fold Change SE	Wald Statistic	P value	Adjusted P value	Significant
1	0.102016124	0.812183269	2.034378736	0.399229138	0.689724375	NA	
2	3.088038856	-0.243922228	1.40861813	-0.173164197	0.862522365	NA	
3	89.22533307	6.621993225	1.124478291	5.888947149	3.89E-09	1.40E-07	*
4	5.636962585	-0.10153496	0.939580992	-0.108064085	0.913944858	NA	
5	0	NA	NA	NA	NA	NA	
6	662.6051047	0.238885039	0.663464391	0.360057061	0.718804462	0.862565355	
7	127.2207271	1.567843124	0.696857336	2.249876759	0.02445677	0.097827079	
8	6004.466082	0.416074083	0.672354224	0.618831664	0.53602726	0.742191591	
9	41.17001542	1.1903704	0.764116308	1.557839281	0.119271352	0.252574627	
10	7.661284373	-1.176160506	1.154679615	-1.018603334	0.308391321	0.462586981	
11	5.684735052	2.085167474	1.173505285	1.77687097	0.075589472	0.194372927	
12	0.051463604	0.069333186	2.034378736	0.034080766	0.972812746	NA	
13	0.078512929	0.812183269	2.034378736	0.399229138	0.689724375	NA	
14	0	NA	NA	NA	NA	NA	
15	0.543390742	0.815260813	1.924622628	0.423595151	0.671861092	NA	
16	3131.008533	1.867761604	0.872772089	2.140033609	0.032352051	0.116467383	
17	0	NA	NA	NA	NA	NA	
18	14.74420332	-2.020323536	1.316775777	-1.534295794	NA	NA	
19	105.5292851	1.896484857	1.073687292	1.766328867	NA	NA	
20	1611.946498	-1.32517235	0.69302603	-1.912153791	0.055856471	0.166767421	
21	3.634538874	0.695765933	1.029836663	0.675608044	0.499289533	NA	
22	332.3101743	1.612355147	0.858015471	1.879167919	0.060221569	0.166767421	
23	8.592376644	0.699007284	0.930281405	0.751393375	0.452415949	0.651478966	
24	4.20889304	0.325176988	1.024964952	0.31725669	0.751048837	NA	
25	395.6306054	-2.36093238	0.715490106	-3.299741478	0.000967739	0.006967723	*
26	14.18201925	0.169050804	1.0081944	0.167676793	0.866837554	0.891604341	
27	17.29408486	2.283432389	0.947832774	2.409108918	0.015991525	0.071961861	
28	21.13412816	-1.13681268	0.825993587	-1.376297223	0.168729632	0.303713338	
29	0	NA	NA	NA	NA	NA	
30	88.29375493	-1.620025007	0.846947323	-1.912781306	0.055776055	0.166767421	
31	0	NA	NA	NA	NA	NA	
32	33.05812767	1.058289671	0.817386292	1.294724028	0.195415448	0.319770733	
33	264.5168175	-0.384134067	0.656649773	-0.584990786	0.558553912	0.744738549	
34	526.7566446	-0.932162847	0.828836208	-1.124664727	0.260731134	0.408100906	
35	305.7155	-2.070818292	0.701650845	-2.951351525	0.003163866	0.018983197	*
36	9.45002256	-0.828407199	1.978962053	-0.418606915	0.675503435	0.838555989	
37	25.39945981	6.332013882	1.883836986	3.361232383	0.000775955	0.006967723	*
38	38.06731438	3.627970498	0.927107277	3.913215427	9.11E-05	0.001092903	*
39	0	NA	NA	NA	NA	NA	
40	0.612096745	2.129651462	2.026409876	1.050948028	0.29328246	NA	
41	292.0199408	3.447745766	0.706565649	4.879583054	1.06E-06	1.91E-05	*
42	17.96133956	1.586422189	1.008673458	1.572780742	0.115769596	0.252574627	
43	0	NA	NA	NA	NA	NA	
44	0	NA	NA	NA	NA	NA	
45	1.292461508	1.653424638	1.621803188	1.019497711	0.307966739	NA	
46	24.74989721	0.238534424	0.895135755	0.26647849	0.789870724	0.891604341	
47	224.0900202	5.265126214	1.297147388	4.059003828	NA	NA	

	Chromium Mid						
#	Mean	Fold Change	Fold Change SE	Wald Statistic	P value	Adjusted P value	Significant
1	0	NA	NA	NA	NA	NA	
2	7.375886073	1.139145662	0.999859536	1.139305693	0.254575675	0.424782464	
3	49.10308723	5.009543829	0.997033958	5.024446551	5.05E-07	2.42E-05	*
4	5.456199173	-0.629179158	0.819980308	-0.767310082	0.442897176	0.630880906	
5	0	NA	NA	NA	NA	NA	
6	1003.082115	0.751268115	0.545087188	1.378253115	0.168125153	0.384286064	
7	132.3058013	1.249801644	0.499862518	2.500290776	0.012409141	0.054148978	
8	6732.099118	0.245811933	0.492572629	0.49903693	0.617753368	0.706003849	
9	55.06371787	1.289269014	0.746849571	1.726276703	0.084297645	0.224793719	
10	8.457461251	-1.810911542	0.895369673	-2.02252946	0.043121683	0.147845771	
11	2.533661512	0.110596429	1.087213072	0.101724705	0.918975192	0.918975192	
12	1.595830382	2.274216655	1.314094401	1.730634157	0.083517036	0.224793719	
13	0.53613552	1.172548836	1.325590688	0.884548185	0.376400363	NA	
14	0.378805878	0.84539267	1.324091398	0.638470026	0.523167759	NA	
15	1.478647482	1.863730043	1.188339122	1.568348638	0.116799791	0.280319498	
16	2320.663519	0.86076394	0.79467368	1.08316654	0.278734509	0.445975214	
17	0	NA	NA	NA	NA	NA	
18	19.4441065	-1.57731517	1.143114688	-1.379839823	NA	NA	
19	238.2016347	2.718604384	0.824688134	3.29652419	0.000978892	0.009397362	*
20	2088.013685	-1.029467351	0.40839518	-2.520762732	0.011710078	0.054148978	
21	3.917701476	0.493145266	0.820557338	0.600988186	0.547847855	0.674274284	
22	438.7304713	1.749627528	0.578986092	3.02188179	0.002512086	0.017225734	*
23	12.11516734	1.081320326	0.801968442	1.348332764	0.177551379	0.387384826	
24	3.141761672	-1.166333298	0.97917137	-1.191143178	0.233597382	0.424782464	
25	552.8911347	-1.384415192	0.534269652	-2.591229331	0.009563374	0.05100466	
26	14.00892525	-0.393273026	0.645042692	-0.609685267	0.542070316	0.674274284	
27	14.91586491	1.638869495	0.739333509	2.216684994	0.026644625	0.098380154	
28	24.69769384	-1.156664735	0.682919477	-1.693705882	0.090321186	0.228179839	
29	0	NA	NA	NA	NA	NA	
30	96.54892417	-2.196229975	0.662197996	-3.316575991	0.000911278	0.009397362	*
31	0	NA	NA	NA	NA	NA	
32	26.34688836	0.097282318	0.639522144	0.152117201	0.879094498	0.911307387	
33	342.076362	-0.114189919	0.53953682	-0.21164435	0.832384507	0.905092333	
34	549.2288929	-1.660146164	0.484499445	-3.426518198	0.000611373	0.009397362	*
35	429.6870054	-1.163803368	0.515622022	-2.257086233	0.024002687	0.096010748	
36	10.3333582	-1.001955433	1.337171998	-0.749309315	NA	NA	
37	0.163583926	0.568185169	1.32352296	0.429297553	0.667706697	NA	
38	39.33107351	3.066551937	0.924338502	3.317563784	0.000908062	0.009397362	*
39	0	NA	NA	NA	NA	NA	
40	0	NA	NA	NA	NA	NA	
41	145.5307688	1.967662572	0.701147582	2.806345799	0.005010689	0.030064133	*
42	18.99793635	1.263799505	0.979861638	1.289773429	NA	NA	
43	0	NA	NA	NA	NA	NA	
44	0	NA	NA	NA	NA	NA	
45	0.452374134	-0.341615482	1.325879519	-0.257651979	0.79667551	NA	
46	36.39794466	0.58182714	0.838409979	0.693964952	0.487704189	0.632697326	
47	11.26022953	0.490400385	0.886975169	0.552890771	0.580338207	0.67942034	

	Chromium Low						
#	Mean	Fold Change	Fold Change SE	Wald Statistic	P value	Adjusted P value	Significant
1	0	NA	NA	NA	NA	NA	
2	7.921656538	1.163598077	0.861109332	1.351277978	0.176606404	0.955697868	
3	0.814612405	-0.93665185	1.042033746	-0.898869018	0.368722432	0.955697868	
4	5.474304706	-0.65693253	0.719021967	-0.913647372	0.360902166	0.955697868	
5	0	NA	NA	NA	NA	NA	
6	723.4075019	-0.02315938	0.508445173	-0.045549415	0.963669389	0.963669389	
7	77.45053221	0.152911214	0.456945781	0.334637543	0.737898521	0.955697868	
8	6884.136288	0.331276537	0.386592061	0.856915003	0.391491861	0.955697868	
9	19.05651555	-1.420526987	0.725260091	-1.958644911	0.050154386	0.812501056	
10	7.663138035	-1.486725335	0.814581829	-1.825139332	0.067980006	0.862374012	
11	1.752829401	-0.584137376	1.062955809	-0.549540603	0.582634508	0.955697868	
12	1.229377641	1.476311018	1.055003371	1.399342465	0.161710311	0.955697868	
13	0.186631919	0.447301784	0.985518446	0.453874593	0.649919091	0.955697868	
14	0	NA	NA	NA	NA	NA	
15	0.360786967	-0.092501293	0.985518956	-0.093860491	0.925219979	0.955697868	
16	3820.918906	1.70796745	0.736493895	2.319051742	0.020392231	0.550590236	
17	0	NA	NA	NA	NA	NA	
18	14.70894855	-1.911401798	1.034513791	-1.847632978	NA	NA	
19	64.41075578	0.663376689	0.735770684	0.901607938	0.367265173	0.955697868	
20	2773.591257	0.131582966	0.380687338	0.345645766	0.729608947	0.955697868	
21	3.209863753	0.074447857	0.807725938	0.0921697	0.926563212	0.955697868	
22	190.8284211	0.056965083	0.458342689	0.124284916	0.90108969	0.955697868	
23	12.30972042	0.969987388	0.83270268	1.164866418	0.244073069	0.955697868	
24	3.870860573	-0.239665224	0.724554685	-0.330775894	0.740813771	0.955697868	
25	687.2995259	-0.253900576	0.421159162	-0.602861338	0.546600938	0.955697868	
26	15.08838613	-0.036544239	0.519315235	-0.07037005	0.943899129	0.955697868	
27	7.483253276	0.273502571	0.843039702	0.324424307	0.74561682	0.955697868	
28	28.25926026	-0.525661663	0.614888767	-0.854889032	0.392612583	0.955697868	
29	0	NA	NA	NA	NA	NA	
30	108.8613568	-0.969099932	0.543409454	-1.783369657	0.074526149	0.862374012	
31	0	NA	NA	NA	NA	NA	
32	23.21926032	-0.270772622	0.603983801	-0.448311066	0.653928715	0.955697868	
33	263.33458	-0.881471663	0.409475912	-2.152682583	0.031343633	0.634708574	
34	510.6934812	-1.585702318	0.423718314	-3.742350199	0.000182307	0.014766884	*
35	521.6031695	-0.184405968	0.436283624	-0.422674512	0.672532754	0.955697868	
36	7.119323655	-1.505748099	1.012133182	-1.487697593	NA	NA	
37	0	NA	NA	NA	NA	NA	
38	4.288378199	-0.820193508	0.881206762	-0.930761704	0.351976843	0.955697868	
39	0	NA	NA	NA	NA	NA	
40	0	NA	NA	NA	NA	NA	
41	66.60481439	0.489568014	0.513917047	0.952620696	0.340782287	0.955697868	
42	6.599352633	-1.087824165	0.977155891	-1.113255495	0.265598715	0.955697868	
43	0	NA	NA	NA	NA	NA	
44	0	NA	NA	NA	NA	NA	
45	0.409456505	-0.328258946	0.987361341	-0.332460805	0.739541331	0.955697868	
46	27.0034018	0.150418772	0.689893691	0.218031812	0.827404325	0.955697868	
47	6.031982539	-1.282599964	0.877046674	-1.462407877	0.143629472	0.955697868	

	Cobalt High						
#	Mean	Fold Change	Fold Change SE	Wald Statistic	P value	Adjusted P value	Significant
1	0	NA	NA	NA	NA	NA	
2	1.013179677	-2.130255949	1.468551728	-1.450582849	0.146896054	0.993748248	
3	392.7571105	0.641759184	1.492857314	0.429886485	0.667278217	0.993748248	
4	21.1519726	1.539833579	0.781739582	1.969752606	0.048866731	0.956051715	
5	0	NA	NA	NA	NA	NA	
6	444.48228	0.048667655	0.825419931	0.058961086	0.952983103	0.993825236	
7	55.64716641	0.984333254	0.828346782	1.188310591	NA	NA	
8	4161.276555	-0.10851265	0.801624656	-0.135365909	0.892322577	0.993748248	
9	3.693262265	0.147466333	1.076985379	0.136925103	0.891089997	0.993748248	
10	3.164721273	-0.0252733	1.302410652	-0.019405017	0.984518009	0.996520186	
11	0.956971943	-0.588910305	1.506044949	-0.391031029	0.695774301	0.993748248	
12	0	NA	NA	NA	NA	NA	
13	0	NA	NA	NA	NA	NA	
14	0	NA	NA	NA	NA	NA	
15	1.292869152	1.551999763	1.433184968	1.082902625	0.278851647	0.993748248	
16	1182.701351	-0.340304439	0.954510544	-0.356522451	0.721449342	0.993748248	
17	0	NA	NA	NA	NA	NA	
18	0.480685793	-1.162106024	1.530072424	-0.759510469	0.447547254	0.993748248	
19	885.9738555	-0.545994408	1.078766911	-0.506128249	0.612766621	0.993748248	
20	1463.527879	-0.264899133	0.793110966	-0.334000088	0.738379492	0.993748248	
21	3.255673237	0.373330357	1.081970391	0.345046741	0.730059232	0.993748248	
22	812.371068	-1.090798907	0.998945118	-1.091950786	0.274854733	0.993748248	
23	10.0267715	-0.201662842	1.095660388	-0.184055976	0.853969542	0.993748248	
24	4.120118167	0.956294107	1.069766245	0.893928101	0.371360362	0.993748248	
25	297.0944516	-1.141919059	0.819908668	-1.392739341	0.163698627	0.993748248	
26	17.44048826	-0.884327491	0.817176763	-1.082174054	0.279175195	0.993748248	
27	20.9591578	0.735123504	0.994759256	0.738996395	0.459909186	0.993748248	
28	19.44474666	0.730934266	1.039986078	0.702830818	0.482161189	0.993748248	
29	0	NA	NA	NA	NA	NA	
30	37.84307142	-0.683694489	0.99410225	-0.68775067	0.491609805	0.993748248	
31	0	NA	NA	NA	NA	NA	
32	28.75803147	0.450380992	0.889396138	0.506389642	0.612583145	0.993748248	
33	238.2615989	1.160606893	0.76195938	1.523187356	0.127711838	0.993748248	
34	558.5298892	2.054056931	0.942915168	2.178411168	0.029375439	0.956051715	
35	312.2107034	-1.505450555	0.907809982	-1.658332233	0.097250426	0.993748248	
36	0.918984285	-1.202167465	1.509356844	-0.796476638	0.425755049	0.993748248	
37	220.7157163	4.415703753	1.133179732	3.896737322	9.75E-05	0.0071173	*
38	3.066976762	-0.24131223	1.167261795	-0.206733597	0.836217917	0.993748248	
39	0	NA	NA	NA	NA	NA	
40	0	NA	NA	NA	NA	NA	
41	269.1895404	0.938390996	0.868822588	1.080072053	0.280110096	0.993748248	
42	8.127799117	1.999960573	1.030936831	1.939944828	0.052386395	0.956051715	
43	0.210255132	0.387873188	1.523734209	0.254554361	0.799067313	0.993748248	
44	0.106936449	0.387873188	1.523734209	0.254554361	0.799067313	0.993748248	
45	0.688099563	1.751967796	1.529157088	1.145708188	0.251915915	0.993748248	
46	46.2453285	0.434462209	0.888824751	0.488805255	0.624979577	0.993748248	
47	221.2986291	3.510738543	1.091895096	3.215270912	NA	NA	

	Cobalt Mid						
#	Mean	Fold Change	Fold Change SE	Wald Statistic	P value	Adjusted P value	Significant
1	0	NA	NA	NA	NA	NA	
2	3.856619908	1.256090702	1.033127876	1.215813387	0.224056018	0.973953231	
3	505.5247689	0.60329195	1.207485322	0.499626736	0.617337928	0.973953231	
4	22.45118741	1.302703185	0.659338316	1.975773518	0.048180434	0.963608681	
5	0	NA	NA	NA	NA	NA	
6	523.6762952	0.132047459	0.680035067	0.194177426	0.846036958	0.973953231	
7	56.17143324	0.656185826	0.662660786	0.990228847	0.322062276	0.973953231	
8	4303.532802	-0.344614978	0.62781235	-0.548913984	0.583064481	0.973953231	
9	3.794903643	0.014764656	1.040814769	0.014185671	0.988681852	0.992441225	
10	2.014529114	-1.905439009	1.154920968	-1.649843636	0.098974921	0.973953231	
11	5.689334649	2.229927266	1.008032837	2.212157366	0.026955794	0.927313509	
12	0	NA	NA	NA	NA	NA	
13	0.072303007	0.140241855	1.17692274	0.119159781	0.90514877	0.973953231	
14	0	NA	NA	NA	NA	NA	
15	0.598315867	0.132243446	1.211542236	0.10915298	0.913081154	0.973953231	
16	2005.964099	0.626977767	0.784215227	0.799497057	0.424002252	0.973953231	
17	0	NA	NA	NA	NA	NA	
18	0.87325475	-0.224978768	1.183493837	-0.190097118	0.849233028	0.973953231	
19	1148.666891	-0.283805018	0.880086216	-0.322474109	0.747093549	0.973953231	
20	1329.099754	-0.647533423	0.585528347	-1.105895943	0.268771561	0.973953231	
21	3.971722621	0.501477941	0.942079195	0.532309751	0.59451148	0.973953231	
22	1255.083055	-0.120491357	0.82565041	-0.145935078	0.88397264	0.973953231	
23	12.93333216	0.298714027	0.843346818	0.354200693	0.723188484	0.973953231	
24	2.513593759	-0.369288455	0.954804401	-0.386768698	0.698927453	0.973953231	
25	314.2727627	-0.869333183	0.659668148	-1.317834105	0.187559188	0.973953231	
26	18.5023772	-0.762392362	0.63895911	-1.193178641	0.232799418	0.973953231	
27	16.74014109	-0.136201286	0.772558508	-0.176298992	0.860059054	0.973953231	
28	17.76419782	0.438781908	0.714077417	0.614473862	0.53890224	0.973953231	
29	0	NA	NA	NA	NA	NA	
30	46.51795175	-0.038358676	0.787200111	-0.048727986	0.961136073	0.985780587	
31	0	NA	NA	NA	NA	NA	
32	18.29269866	-1.602658191	0.759201974	-2.110977375	0.034774257	0.927313509	
33	209.989264	0.691908239	0.608996675	1.136144525	0.255896083	0.973953231	
34	211.839163	0.05615691	0.666372006	0.084272613	0.932839686	0.981936512	
35	329.9023609	-1.117778507	0.647799068	-1.725501876	0.084437066	0.973953231	
36	0.955192932	-1.371548063	1.216369595	-1.127575096	0.259499409	0.973953231	
37	98.17810399	2.849156855	0.991304113	2.874150139	0.004051163	0.324093064	
38	2.317534473	-1.306410528	1.085714241	-1.203272905	0.228870731	0.973953231	
39	0	NA	NA	NA	NA	NA	
40	0	NA	NA	NA	NA	NA	
41	234.8848894	0.177436993	0.752510113	0.2357935	0.813592913	0.973953231	
42	5.302033946	0.736099132	1.040282097	0.707595693	0.479196368	0.973953231	
43	0	NA	NA	NA	NA	NA	
44	0	NA	NA	NA	NA	NA	
45	1.273333842	1.940281428	1.216805829	1.594569472	0.110808544	0.973953231	
46	53.99657264	0.657157775	0.722439611	0.909636966	0.363013997	0.973953231	
47	28.48299801	-0.380139901	0.757210717	-0.502026573	0.615648829	0.973953231	

	Cobalt Low						
#	Mean	Fold Change	Fold Change SE	Wald Statistic	P value	Adjusted P value	Significant
1	0	NA	NA	NA	NA	NA	
2	3.397977356	0.887043795	0.921636775	0.962465712	0.335815719	0.968893609	
3	175.625397	-1.699494871	1.048111947	-1.621482205	0.104914263	0.968893609	
4	9.993224712	-0.598701445	0.713849733	-0.83869394	0.40164108	0.968893609	
5	0	NA	NA	NA	NA	NA	
6	500.9389617	-0.070110263	0.522457106	-0.134193337	0.893249694	0.968893609	
7	37.95819528	-0.407233894	0.526696689	-0.773184837	0.439412998	0.968893609	
8	4551.055092	-0.238039415	0.524110455	-0.454177955	0.649700748	0.968893609	
9	7.489571869	1.156460085	0.794208593	1.45611631	0.14536047	0.968893609	
10	16.65946259	1.481516919	1.047365372	1.414517759	NA	NA	
11	2.515940432	0.733707396	1.049168714	0.699322603	0.484350445	0.968893609	
12	0.515935403	0.792682412	0.972855687	0.814799587	0.415187037	0.968893609	
13	0	NA	NA	NA	NA	NA	
14	0	NA	NA	NA	NA	NA	
15	0.63292822	0.267003223	1.046749388	0.255078461	0.7986625	0.968893609	
16	1548.552025	-0.003872018	0.76716456	-0.005047181	0.995972949	0.995972949	
17	0	NA	NA	NA	NA	NA	
18	1.018199107	-0.291478856	1.037897794	-0.280835798	0.778836345	0.968893609	
19	778.5678655	-1.586087742	0.924219651	-1.716137219	0.086136931	0.968893609	
20	2263.500459	0.593576141	0.606753705	0.978281857	0.327934941	0.968893609	
21	5.410593918	1.060565013	0.723633207	1.465611309	0.142754209	0.968893609	
22	863.3437578	-1.295772368	0.856161266	-1.513467636	0.130160916	0.968893609	
23	15.83210961	0.536579718	0.957132958	0.560611474	0.575062428	0.968893609	
24	4.107831905	0.589655302	0.737863493	0.79913874	0.424209969	0.968893609	
25	572.3182504	0.615321611	0.643520997	0.956179539	0.338981535	0.968893609	
26	21.71701217	-0.459864392	0.521426416	-0.881935356	0.377811766	0.968893609	
27	22.50039046	0.470867778	0.759725974	0.619786337	0.535398465	0.968893609	
28	26.64863735	1.025082114	0.72002538	1.423674975	0.154540581	0.968893609	
29	0	NA	NA	NA	NA	NA	
30	80.70880062	0.966284932	0.645509543	1.496933612	0.134410533	0.968893609	
31	0	NA	NA	NA	NA	NA	
32	36.13707993	0.516579061	0.71639931	0.721076993	0.470862145	0.968893609	
33	232.3572215	0.78407825	0.592349382	1.323675307	0.185610903	0.968893609	
34	423.5426425	1.26839538	0.717269362	1.768366876	0.07699959	0.968893609	
35	514.373788	0.093266102	0.704828891	0.132324459	0.894727662	0.968893609	
36	0.828907315	-1.12120021	0.99318516	-1.128893439	0.258942789	0.968893609	
37	12.67609235	-0.285624886	0.974486852	-0.293102863	0.769443529	0.968893609	
38	4.546362492	0.310981407	0.871588237	0.356798536	0.721242632	0.968893609	
39	0	NA	NA	NA	NA	NA	
40	0	NA	NA	NA	NA	NA	
41	165.9808769	-0.81773082	0.71620528	-1.141754806	0.253555953	0.968893609	
42	3.756463943	0.162032169	0.815085398	0.198791648	0.84242573	0.968893609	
43	0	NA	NA	NA	NA	NA	
44	0	NA	NA	NA	NA	NA	
45	0.107270739	0.095794485	0.966990975	0.099064508	0.921087052	0.968893609	
46	39.60408112	-0.22206522	0.677493986	-0.327774453	0.743082205	0.968893609	
47	25.73636242	-0.75259361	0.74576912	-1.009150942	0.31290225	0.968893609	

	Cadmium High						
#	Mean	Fold Change	Fold Change SE	Wald Statistic	P value	Adjusted P value	Significant
1	0	NA	NA	NA	NA	NA	
2	2.65315986	-0.69980792	1.011113405	-0.692116153	0.488864388	NA	
3	255.2048268	4.950861459	1.226638384	4.036121421	5.43E-05	0.000434737	*
4	7.671705787	0.823411242	0.704043658	1.169545713	0.242183835	0.387494136	
5	0	NA	NA	NA	NA	NA	
6	654.1064681	1.013677232	0.506262768	2.002274899	0.045255174	0.111397352	
7	70.07085394	1.342061859	0.741486847	1.809960439	0.070301922	0.160690108	
8	4358.857556	-0.655635044	0.471312325	-1.391084022	0.164199949	0.276547283	
9	11.59902327	0.79244629	0.859092445	0.922422604	0.356308184	0.507187802	
10	2.392866488	-1.216786872	1.28783617	-0.944830484	0.344745426	NA	
11	1.553961062	-0.079344443	1.054434668	-0.075248326	0.940017135	NA	
12	0	NA	NA	NA	NA	NA	
13	0	NA	NA	NA	NA	NA	
14	0	NA	NA	NA	NA	NA	
15	1.465576532	-1.884730353	1.313441165	-1.434956055	0.15129962	NA	
16	1888.631205	-1.633779402	0.984632882	-1.659277717	0.097059843	0.182700881	
17	0	NA	NA	NA	NA	NA	
18	5.046967434	-0.718394263	0.745000938	-0.964286388	0.334902363	NA	
19	441.9372539	-0.239553809	1.215693878	-0.197051094	0.843787554	0.871006507	
20	2010.287684	-1.50408581	0.474342136	-3.170888046	0.001519737	0.009726317	*
21	6.401524819	0.813372622	0.919677571	0.884410633	0.376474585	NA	
22	191.4151445	-3.176394537	1.005457639	-3.159153022	NA	NA	
23	8.089483057	-0.932507462	1.136243709	-0.820693179	0.411821057	0.54184144	
24	3.816305467	0.04618591	1.039371216	0.044436395	0.964556551	NA	
25	491.3345124	-1.227823211	0.58704756	-2.091522552	0.036481247	0.097283325	
26	19.76823914	-0.116608082	0.549961994	-0.212029346	0.83208414	0.871006507	
27	29.89945985	0.727522144	0.723560365	1.005475396	0.314667991	0.479494081	
28	16.37004756	-0.420068216	0.541901401	-0.775174627	0.438236479	0.54184144	
29	0	NA	NA	NA	NA	NA	
30	148.0744592	0.715992068	0.927718145	0.771777584	0.44024617	0.54184144	
31	0	NA	NA	NA	NA	NA	
32	72.79847957	2.16146806	0.844921908	2.558186785	0.010521956	0.054271326	
33	381.688449	0.402565145	0.678727537	0.593117448	0.553102554	0.61032006	
34	376.6211086	0.384620109	0.611597371	0.628877962	0.529428955	0.605061662	
35	453.6445827	-2.876794303	0.50884083	-5.653623162	1.57E-08	5.03E-07	*
36	0.846537188	1.879330182	1.443037868	1.302342942	0.192799178	NA	
37	22.87423756	6.373952137	1.175826836	5.420825535	5.93E-08	9.49E-07	*
38	12.8483326	1.684519956	0.669541478	2.515930695	0.011871853	0.054271326	
39	0	NA	NA	NA	NA	NA	
40	0	NA	NA	NA	NA	NA	
41	104.5083439	1.326465605	0.598756098	2.215368843	0.026734758	0.088904822	
42	8.516645504	0.0303698	1.29915885	0.02337651	0.981349942	0.981349942	
43	0	NA	NA	NA	NA	NA	
44	0.071081835	0.149696116	1.856895055	0.080616357	0.935747057	NA	
45	0.213245505	0.747584517	1.856177786	0.402754802	0.687128614	NA	
46	48.00835503	-1.396850068	0.624951424	-2.235133828	0.025408564	0.088904822	
47	928.587952	4.203351602	0.982535339	4.278066585	1.89E-05	0.000201092	*

	Cadmium Mid						
#	Mean	Fold Change	Fold Change SE	Wald Statistic	P value	Adjusted P value	Significant
1	0	NA	NA	NA	NA	NA	
2	2.295124884	-0.52158215	1.200044174	-0.434635792	0.663826811	0.992655411	
3	591.6999607	6.340931466	1.116124999	5.681201903	1.34E-08	1.30E-06	*
4	7.218565501	0.95957101	0.789609787	1.215247108	0.224271858	0.992655411	
5	0	NA	NA	NA	NA	NA	
6	639.0020324	1.230350524	0.714135826	1.722852262	0.084915245	0.74879807	
7	61.61948468	1.36810016	0.750336015	1.823316664	0.068255444	0.662077805	
8	4677.56676	0.083766519	0.618504802	0.135433903	0.89226882	0.992655411	
9	9.171315433	0.660372355	1.002732796	0.658572611	0.510170256	0.992655411	
10	2.103577097	-1.094090069	1.393472316	-0.785153789	0.432363406	0.992655411	
11	1.098117761	-0.525355636	1.188848608	-0.44190289	0.658559479	0.992655411	
12	0	NA	NA	NA	NA	NA	
13	0.061888688	0.296214511	1.708578508	0.173368979	0.862361407	0.992655411	
14	0	NA	NA	NA	NA	NA	
15	1.647391891	-0.703942953	1.28810128	-0.546496587	0.584724633	0.992655411	
16	2403.696237	-0.131933559	0.838712791	-0.157304813	0.875004627	0.992655411	
17	0.240417293	0.953308023	1.707597662	0.558274378	0.576657038	0.992655411	
18	6.152071597	0.258262477	0.76721588	0.336622955	0.736401149	0.992655411	
19	696.3978634	1.272684352	1.083290669	1.174831824	0.240062005	0.992655411	
20	1721.288801	-1.555686955	0.62139425	-2.503542566	0.012295689	0.170383118	
21	4.245075404	0.292160066	1.108032079	0.263674736	0.792030564	0.992655411	
22	206.3554932	-0.955791584	0.949718438	-1.006394681	0.314225751	0.992655411	
23	8.291784867	-0.389753807	1.039893903	-0.374801512	0.70780809	0.992655411	
24	3.410551575	-0.014218627	1.054807748	-0.013479828	0.989244979	0.992655411	
25	404.0081658	-1.459584799	0.696924352	-2.094323142	0.036231204	0.439303351	
26	17.56057858	-0.034791739	0.656685163	-0.052980852	0.957747164	0.992655411	
27	24.8516184	0.67352888	0.725926167	0.927820089	0.353500902	0.992655411	
28	12.81011285	-0.84880877	0.745238196	-1.138976471	0.254712968	0.992655411	
29	0	NA	NA	NA	NA	NA	
30	97.54273926	-0.101284597	0.818940635	-0.123677582	0.901570563	0.992655411	
31	0	NA	NA	NA	NA	NA	
32	19.81543529	-0.053417226	0.760232563	-0.070264323	0.943983279	0.992655411	
33	296.8542771	0.160789665	0.649485086	0.247564831	0.804471125	0.992655411	
34	230.5203969	-0.669597278	0.676755505	-0.989422728	0.322456359	0.992655411	
35	501.4358396	-1.097270433	0.799740374	-1.372033311	0.170053068	0.992655411	
36	0.437102202	1.067449391	1.647132326	0.648065353	0.516942682	0.992655411	
37	17.54822633	5.975448844	1.208171731	4.945860503	7.58E-07	3.68E-05	*
38	5.562065535	0.165254521	0.860295955	0.192090316	0.847671461	0.992655411	
39	0	NA	NA	NA	NA	NA	
40	0	NA	NA	NA	NA	NA	
41	162.2644752	2.265477473	0.86078977	2.631859198	0.008491905	0.149453956	
42	5.242475448	-1.153735931	1.116841914	-1.033034234	0.301587878	0.992655411	
43	0	NA	NA	NA	NA	NA	
44	0	NA	NA	NA	NA	NA	
45	0.206758118	0.63291064	1.708366526	0.370477079	0.711027052	0.992655411	
46	52.69724908	-0.3559162	0.776797674	-0.458183915	0.646820315	0.992655411	
47	463.1636615	3.336387799	1.046247921	3.188907459	0.001428116	0.03463181	*

	Cadmium Low						
#	Mean	Fold Change	Fold Change SE	Wald Statistic	P value	Adjusted P value	Significant
1	0	NA	NA	NA	NA	NA	
2	1.680124279	-1.680278841	1.201335753	-1.398675463	0.161910325	0.907686383	
3	313.8219292	5.679002603	1.047722318	5.420331803	5.95E-08	2.62E-06	*
4	8.13758711	1.380273001	0.712701681	1.936677067	0.052784831	0.516118346	
5	0	NA	NA	NA	NA	NA	
6	323.9931684	-0.200224194	0.630870551	-0.31737762	0.750957087	0.963109772	
7	39.15723658	0.586018019	0.689298138	0.850166258	0.395232658	0.963109772	
8	5035.468995	0.41056429	0.511145399	0.803224075	0.421845233	0.963109772	
9	5.716693305	-0.644996733	1.009064537	-0.639202657	0.522691104	0.963109772	
10	1.359116804	-2.59133162	1.512419669	-1.713368103	0.086644841	0.635395497	
11	2.110190603	1.085375168	1.027123399	1.056713506	0.290642359	0.963109772	
12	0	NA	NA	NA	NA	NA	
13	0	NA	NA	NA	NA	NA	
14	0	NA	NA	NA	NA	NA	
15	1.18638344	-1.427754792	1.328030882	-1.07509156	0.282333738	0.963109772	
16	1590.066453	-0.967758773	0.78294108	-1.236055685	0.216437865	0.963109772	
17	0	NA	NA	NA	NA	NA	
18	4.783278256	-0.068799266	0.832332945	-0.082658348	0.934123205	0.970021505	
19	566.3363845	0.832750816	1.040561749	0.800289667	0.423542988	0.963109772	
20	1659.724048	-1.217100784	0.539258005	-2.256991592	0.0240086	0.301822402	
21	4.406706758	0.345805981	0.968934534	0.356893029	0.721171888	0.963109772	
22	443.3623746	1.004192651	0.884260941	1.13562932	0.256111733	0.963109772	
23	7.317529098	-0.496252207	0.913551683	-0.543211967	0.586983868	0.963109772	
24	1.857052556	-1.265482314	1.311527824	-0.964891701	0.334599058	0.963109772	
25	355.9917923	-1.395665148	0.600778564	-2.323094118	0.020174097	0.301822402	
26	15.84432006	-0.103932081	0.700304281	-0.148409889	0.882019296	0.963109772	
27	26.40992616	0.832673964	0.866704165	0.960736082	0.336684884	0.963109772	
28	16.6132942	0.053878876	0.662618799	0.081312025	0.935193811	0.970021505	
29	0	NA	NA	NA	NA	NA	
30	66.84536815	-1.084229504	0.842286582	-1.287245372	0.198008777	0.963109772	
31	0	NA	NA	NA	NA	NA	
32	33.6622995	1.2248931	0.681126051	1.798335416	0.072123871	0.576990965	
33	275.2558892	0.107008082	0.529854404	0.201957521	0.839949933	0.963109772	
34	210.2240688	-0.658143677	0.558195822	-1.179055183	0.238376203	0.963109772	
35	381.349496	-2.558948782	0.495590361	-5.163435335	2.42E-07	7.11E-06	*
36	0.326978061	0.856094618	1.75610154	0.487497219	0.625906013	0.963109772	
37	119.6523164	8.681199826	1.190476882	7.292203618	3.05E-13	2.68E-11	*
38	6.620883882	0.728678481	0.763545951	0.954334811	0.339914192	0.963109772	
39	0	NA	NA	NA	NA	NA	
40	0	NA	NA	NA	NA	NA	
41	88.47253799	1.360435153	0.594879257	2.286909715	0.022201093	0.301822402	
42	15.97414923	1.555244911	1.073807325	1.448346342	0.147520213	0.907686383	
43	0	NA	NA	NA	NA	NA	
44	0	NA	NA	NA	NA	NA	
45	0	NA	NA	NA	NA	NA	
46	47.64564762	-0.515815585	0.626854665	-0.82286312	0.410585837	0.963109772	
47	58.63983976	0.305233379	0.906602586	0.336678257	0.736359456	0.963109772	

	Arsenic High						
#	Mean	Fold Change	Fold Change SE	Wald Statistic	P value	Adjusted P value	Significant
1	0	NA	NA	NA	NA	NA	
2	11.70996567	-5.502436247	1.198668957	-4.590455283	4.42E-06	4.42E-05	*
3	16.73126381	-3.531537451	0.880785551	-4.009531542	6.08E-05	0.00052148	*
4	25.32799691	1.521956599	0.868497267	1.75240229	NA	NA	
5	0	NA	NA	NA	NA	NA	
6	1783.450981	2.158506776	0.683431571	3.158336352	0.001586724	0.00732334	*
7	481.2969607	3.490078456	0.626523932	5.570542925	2.54E-08	3.05E-07	*
8	5137.207634	-0.975063041	0.691981272	-1.409088773	0.15880892	0.321919271	
9	6.155417357	-1.499594465	1.408029992	-1.0650302	0.286862263	0.430293395	
10	1.993892249	-3.477158119	1.469600145	-2.366057278	0.017978663	0.053657091	
11	12.61437287	1.155767415	1.290213874	0.895795215	0.370362146	0.52908878	
12	0.933718172	0.023241724	2.135469916	0.010883658	0.991316269	0.991316269	
13	0	NA	NA	NA	NA	NA	
14	0	NA	NA	NA	NA	NA	
15	0.561151536	-0.130389298	1.926908886	-0.067667599	0.946050242	NA	
16	778.8268854	-4.77957719	0.733102619	-6.519656412	7.05E-11	1.06E-09	*
17	0.079019318	0.403767608	2.212926551	0.182458658	0.855222796	NA	
18	1.853886422	0.141422391	1.282663342	0.110256828	0.912205695	0.977363245	
19	207.9282739	-7.625872286	0.711788933	-10.71367077	8.78E-27	5.27E-25	*
20	2404.204058	-1.370072455	0.531753556	-2.576517712	0.009980109	0.033267031	*
21	7.450162916	-0.469678761	0.89365687	-0.525569462	0.599187379	0.764920058	
22	373.6396818	-1.961216703	0.517825664	-3.787407307	0.000152227	0.000913365	*
23	10.18356161	-2.179811673	1.197345866	-1.82053635	0.068677366	0.164825678	
24	9.57290474	1.265072876	1.111553684	1.138112261	0.255073609	0.402747804	
25	709.6360721	0.016125376	0.477577812	0.033764919	0.973064611	0.991316269	
26	44.2308324	0.783096035	0.564540957	1.387137681	0.165399784	0.321919271	
27	73.49043623	2.158372597	0.733858086	2.941130771	0.003270164	0.013080658	*
28	32.52664579	0.674196897	0.676775834	0.996189377	0.31915814	0.467060692	
29	0	NA	NA	NA	NA	NA	
30	141.7047169	1.797198989	0.764807919	2.349869744	0.018779982	0.053657091	
31	0	NA	NA	NA	NA	NA	
32	192.8598075	3.68710134	0.529870151	6.958499803	3.44E-12	6.88E-11	*
33	827.2583079	1.648453279	0.469425317	3.511641194	0.000445349	0.002429175	*
34	917.6978598	2.179993164	0.736001762	2.961940141	0.003057072	0.013080658	*
35	603.7513908	-1.120405836	0.760298885	-1.473638669	0.140578904	0.301240509	
36	0.717564991	-2.171013049	2.191559212	-0.990624865	0.321868791	NA	
37	2.986125445	-4.494533532	1.562687612	-2.876156116	0.004025506	0.015095646	*
38	24.04156678	-2.357356201	1.415114142	-1.665841737	0.095744941	0.212766535	
39	0	NA	NA	NA	NA	NA	
40	0	NA	NA	NA	NA	NA	
41	191.0288918	0.256862276	0.97322482	0.263929023	0.791834611	0.912560123	
42	482.2136541	6.464963265	0.873594176	7.400419372	1.36E-13	4.07E-12	*
43	0.550999349	1.912537441	2.195717036	0.871030925	0.383737268	NA	
44	0.190283416	0.807790871	2.212926551	0.365032843	0.71508689	NA	
45	2.004944716	3.708851158	1.874368777	1.978720091	0.04784753	0.119618824	
46	85.63390534	0.075337847	0.674251837	0.111735471	0.911033154	0.977363245	
47	17.16568311	-4.172203519	1.069829847	-3.899875791	9.62E-05	0.000641614	*

	Arsenic Mid						
#	Mean	Fold Change	Fold Change SE	Wald Statistic	P value	Adjusted P value	Significant
1	0	NA	NA	NA	NA	NA	
2	9.731320206	-3.792654293	1.076367576	-3.523567948	0.000425778	0.008533945	*
3	13.17238113	-3.410517677	1.06696947	-3.196452921	0.001391285	0.019477994	*
4	7.700786503	-0.802542319	0.751973571	-1.067248039	0.285859845	0.922659243	
5	0	NA	NA	NA	NA	NA	
6	491.8474905	-0.045682827	0.577860907	-0.079055058	0.93698883	0.995911163	
7	175.907862	2.245746597	0.646666891	3.472802812	0.000515054	0.008533945	*
8	3934.25216	-1.11980657	0.759658009	-1.474093021	0.140456547	0.651811023	
9	25.32217168	2.392364075	0.924897201	2.586627002	0.009692045	0.118727546	
10	3.382430311	-0.006566863	1.281434069	-0.00512462	0.995911163	0.995911163	
11	13.94685187	1.669174132	1.10151921	1.515338196	0.12968678	0.635465223	
12	2.323910131	1.854470703	1.521611566	1.218754342	0.222937446	0.789596963	
13	0	NA	NA	NA	NA	NA	
14	0	NA	NA	NA	NA	NA	
15	0.571545683	0.313416004	1.76057979	0.178018631	0.858708344	0.983737513	
16	800.720092	-1.561333597	0.623267429	-2.505078114	0.012242435	0.133306517	
17	0	NA	NA	NA	NA	NA	
18	15.19991377	3.871380417	1.116007592	3.468955271	0.000522486	0.008533945	*
19	164.2167198	-7.816646778	0.773183379	-10.10969324	5.00E-24	4.90E-22	*
20	2538.88865	-0.277216743	0.621893635	-0.445762309	0.65576895	0.983737513	
21	8.05652999	0.349283903	1.011393776	0.345349073	0.729831958	0.983737513	
22	713.8334823	0.96296583	0.772768101	1.246125234	0.212718422	0.789596963	
23	14.09306876	0.060875121	1.294506609	0.047025732	NA	NA	
24	6.467107642	0.764643665	1.154245338	0.662461991	0.507675186	0.983737513	
25	528.2513872	-0.25931444	0.610900432	-0.424479059	0.671216474	0.983737513	
26	23.22021835	-0.263104073	0.609404552	-0.431739593	0.665930688	0.983737513	
27	36.95632832	1.315501037	0.827272488	1.590166547	0.111797269	0.576638544	
28	46.5978321	1.763978525	0.786784233	2.242010515	0.024960693	0.222377081	
29	0	NA	NA	NA	NA	NA	
30	86.36943483	1.226894265	0.682416853	1.797866304	0.072198197	0.471694888	
31	0	NA	NA	NA	NA	NA	
32	27.2688364	0.560662757	0.691572236	0.810707439	0.4175337	0.983737513	
33	545.3510621	1.263982065	0.602739171	2.097063086	0.035987991	0.293901928	
34	539.9259609	1.599334401	0.838596154	1.907156853	0.056500279	0.395501953	
35	482.7782023	-1.194265027	0.686578545	-1.739444139	0.08195667	0.499761006	
36	0.657944416	-1.065625643	1.822584498	-0.58467832	0.558764034	0.983737513	
37	2.42918646	-3.523812541	1.520057925	-2.318209381	0.020437944	0.200291856	
38	18.49015576	-2.238916967	1.306935096	-1.713104938	0.086693236	0.499761006	
39	0	NA	NA	NA	NA	NA	
40	0	NA	NA	NA	NA	NA	
41	187.0131906	0.708343177	0.722944558	0.979802904	0.327183419	0.983737513	
42	5.203427056	-1.324958152	0.92712894	-1.429098041	0.152976056	0.651811023	
43	0	NA	NA	NA	NA	NA	
44	0.468487228	0.963907257	1.832688548	0.525952573	0.59892116	0.983737513	
45	0.166115901	1.20567655	1.831431257	0.658324764	0.510329469	0.983737513	
46	73.55273415	0.277309895	0.619717424	0.44747797	0.65452999	0.983737513	
47	12.86653422	-4.811618208	0.991770348	-4.851544732	1.23E-06	6.00E-05	*

	Arsenic Low						
#	**Mean**	**Fold Change**	**Fold Change SE**	**Wald Statistic**	**P value**	**Adjusted P value**	**Significant**
1	0	NA	NA	NA	NA	NA	
2	15.92996673	-2.42206662	1.124441091	-2.154018241	0.031238741	0.21867119	
3	36.05907727	-0.127195781	0.952950134	-0.133475799	0.893817102	0.993100019	
4	12.57313747	-0.610839967	0.691709819	-0.883087027	0.377189253	0.754378506	
5	0	NA	NA	NA	NA	NA	
6	838.8705992	0.277634758	0.542951614	0.511343463	0.609110576	0.905869117	
7	228.2421855	1.98455986	0.680952115	2.914389744	0.003563847	0.058209496	
8	4958.232946	-2.218390639	0.678080807	-3.271572673	0.001069511	0.026203019	*
9	22.61740529	1.468049007	1.064058172	1.379669876	0.167688312	0.514657649	
10	2.219819951	-4.180776007	1.428965257	-2.925736638	0.003436417	0.058209496	
11	5.741390692	-1.912320463	1.158422688	-1.650796797	0.098780074	0.407974724	
12	0.726461805	-0.944964957	1.863090392	-0.507202957	0.61201242	0.905869117	
13	0.120576459	0.320516869	1.87174855	0.171239277	0.864035625	0.973281508	
14	0	NA	NA	NA	NA	NA	
15	0.869284901	0.432209562	1.667398495	0.259211918	0.795471736	0.950685733	
16	1500.418454	-0.65282945	0.466487336	-1.399458033	0.161675675	0.514657649	
17	0	NA	NA	NA	NA	NA	
18	2.726701455	0.560643989	1.276663835	0.439147702	0.660554517	0.905869117	
19	270.4700318	-3.311862023	0.919752911	-3.600817113	0.000317219	0.015543715	*
20	3535.830713	-0.582358486	0.439965949	-1.323644451	0.185621155	0.523874003	
21	11.7807843	0.313450248	0.680968917	0.460300376	0.645300631	0.905869117	
22	1135.849234	1.07830368	0.520318149	2.072392982	0.038228807	0.23119391	
23	14.51603103	-1.231828766	1.054741985	-1.16789583	0.242848786	0.566647168	
24	8.03292859	0.307286777	1.097105403	0.280088655	0.779409488	0.942989257	
25	751.0274447	-0.471177778	0.486948592	-0.96761298	0.333237692	0.710824944	
26	38.74362933	-0.01583508	0.467194576	-0.033893973	0.972961699	0.993100019	
27	84.27067796	2.061397613	0.741528897	2.779928903	0.00543708	0.076119121	
28	45.50661895	0.93705073	0.592625562	1.581185136	0.113835713	0.413181477	
29	0	NA	NA	NA	NA	NA	
30	156.7511765	1.569891	0.624757224	2.512801677	0.011977665	0.093097546	
31	0	NA	NA	NA	NA	NA	
32	60.21611745	1.391793478	0.542764683	2.564266839	0.010339402	0.092114673	
33	625.584263	0.674271234	0.454919213	1.482177964	0.138292948	0.484025319	
34	557.6887882	0.904728003	0.538717558	1.679410649	0.093072039	0.407974724	
35	752.4306206	-1.083955675	0.538035988	-2.014652735	0.043941048	0.238388962	
36	0.902862442	-2.290043643	1.864114388	-1.228488798	0.219263532	0.537195654	
37	3.686475887	-4.770123276	1.436316574	-3.321080716	0.000896696	0.026203019	*
38	27.22660804	-2.560056714	1.225550899	-2.088902809	0.036716473	0.23119391	
39	0	NA	NA	NA	NA	NA	
40	0	NA	NA	NA	NA	NA	
41	261.3820072	0.522499023	0.612483188	0.853083045	0.393613253	0.771481976	
42	6.56513254	-2.583300414	0.986040472	-2.619872598	0.008796262	0.08620337	
43	0	NA	NA	NA	NA	NA	
44	0	NA	NA	NA	NA	NA	
45	0.384549532	1.110812981	1.866276408	0.59520282	0.551707906	0.905869117	
46	83.10998839	-0.596636604	0.558179924	-1.068896565	0.28511628	0.649799893	
47	20.15982866	-4.264417943	0.77766037	-5.483650845	4.17E-08	4.08E-06	*
